# Light-driven nanoscale vectorial currents

**DOI:** 10.1038/s41586-024-07037-4

**Published:** 2024-02-07

**Authors:** Jacob Pettine, Prashant Padmanabhan, Teng Shi, Lauren Gingras, Luke McClintock, Chun-Chieh Chang, Kevin W. C. Kwock, Long Yuan, Yue Huang, John Nogan, Jon K. Baldwin, Peter Adel, Ronald Holzwarth, Abul K. Azad, Filip Ronning, Antoinette J. Taylor, Rohit P. Prasankumar, Shi-Zeng Lin, Hou-Tong Chen

**Affiliations:** 1 Center for Integrated Nanotechnologies, Los Alamos National Laboratory, Los Alamos, NM USA; 2 Menlo Systems, Martinsried, Germany; 3 Department of Physics, University of California, Davis, Davis, CA USA; 4 Fu Foundation School of Engineering and Applied Science, Columbia University, New York, NY USA; 5 Center for Integrated Nanotechnologies, Sandia National Laboratories, Albuquerque, NM USA; 6 Institute for Materials Science, Los Alamos National Laboratory, Los Alamos, NM USA; 7 Intellectual Ventures, Bellevue, WA USA

**Keywords:** Metamaterials, Nanophotonics and plasmonics, Electronic properties and devices

## Abstract

Controlled charge flows are fundamental to many areas of science and technology, serving as carriers of energy and information, as probes of material properties and dynamics^1^ and as a means of revealing^2,3^ or even inducing^4,5^ broken symmetries. Emerging methods for light-based current control^5–16^ offer particularly promising routes beyond the speed and adaptability limitations of conventional voltage-driven systems. However, optical generation and manipulation of currents at nanometre spatial scales remains a basic challenge and a crucial step towards scalable optoelectronic systems for microelectronics and information science. Here we introduce vectorial optoelectronic metasurfaces in which ultrafast light pulses induce local directional charge flows around symmetry-broken plasmonic nanostructures, with tunable responses and arbitrary patterning down to subdiffractive nanometre scales. Local symmetries and vectorial currents are revealed by polarization-dependent and wavelength-sensitive electrical readout and terahertz (THz) emission, whereas spatially tailored global currents are demonstrated in the direct generation of elusive broadband THz vector beams^17^. We show that, in graphene, a detailed interplay between electrodynamic, thermodynamic and hydrodynamic degrees of freedom gives rise to rapidly evolving nanoscale driving forces and charge flows under the extremely spatially and temporally localized excitation. These results set the stage for versatile patterning and optical control over nanoscale currents in materials diagnostics, THz spectroscopies, nanomagnetism and ultrafast information processing.

## Abstract (web summary)

Vectorial optoelectronic metasurfaces are described, showing that light pulses can be used to drive and direct local charge flows around symmetry-broken plasmonic nanostructures, leading to tunable responses in terahertz emission.

## Main

Recent advances in controlling photocurrents (currents induced by light fields) have been responsible for a variety of insights in areas ranging from materials characterization and device physics^1^ to electrolytic chemistry^18^ and ultrafast electron diffraction and imaging^19^. The evolution from voltage-driven to light-driven processes has been particularly prominent in information science and microelectronics, in which optoelectronic and optospintronic currents in emerging topological^5,11,12^, magnetic^13,14^ and low-dimensional^15,20^ materials are introducing faster speed limits and light-based control degrees of freedom. However, the broken spatial or temporal symmetries responsible for photocurrent generation in these materials^3^ are typically either intrinsic to the lattice and thus constrained to specific light–matter interaction geometries or otherwise dependent on applied static fields, both of which are difficult to pattern on small length scales. Although coherent lightwave interactions in strong, few-cycle and/or phase-stabilized light fields^6–10^ transcend many material-specific challenges and offer a high degree of control over charge motion, such approaches have also remained limited to single laser spots or larger, micrometre-scale structured light fields.

Plasmonic systems can be used to overcome these spatial limitations by concentrating light down into deeply subdiffractive nanometre scales. Such systems are already known to provide exceptional control over energy flow between photonic, electronic and thermal degrees of freedom on ultrafast timescales^21^. A better understanding of momentum flow in plasmon-excited hot-carrier distributions is also emerging^22^, as determined by spatially tailored plasmonic hotspots and directionality imposed by nanoscale geometry. Although such effects have primarily been explored through nonlinear photoemission into free space^23–25^, linear photocurrent responses under continuous-wave excitation have been observed recently in hybrid plasmonic systems serving as bias-free mid-infrared photodetectors^26,27^, as well as spin-valley-polarized valleytronic transistors^28^.

Here we show that plasmonic metasurfaces offer far broader possibilities for harnessing charge flow, introducing unprecedented capabilities for patterning and actively controlling vectorial currents at nanometre spatial scales and femtosecond timescales. Photocurrents are a universal manifestation of broken inversion symmetry^3^ and we find that asymmetric gold nanoantennas on graphene exhibit strong, light-driven directional responses. Local vectorial current directionality is determined by nanoantenna orientation and locally patterned symmetry, with general implications for optically controlled and global spatially varying vectorial photocurrents. As an immediate application, we demonstrate that these vectorial optoelectronic metasurfaces serve as efficient and versatile sources of ultrafast terahertz (THz) radiation, including broadband THz vector beams. Electrostatic gating and multiphysics modelling reveal a local photothermoelectric driving mechanism and clarify previously unexplored dynamics occurring at the intersection of femtosecond excitation and nanoscale localization.

## Ultrafast directional photocurrent

**Fig. 1:**
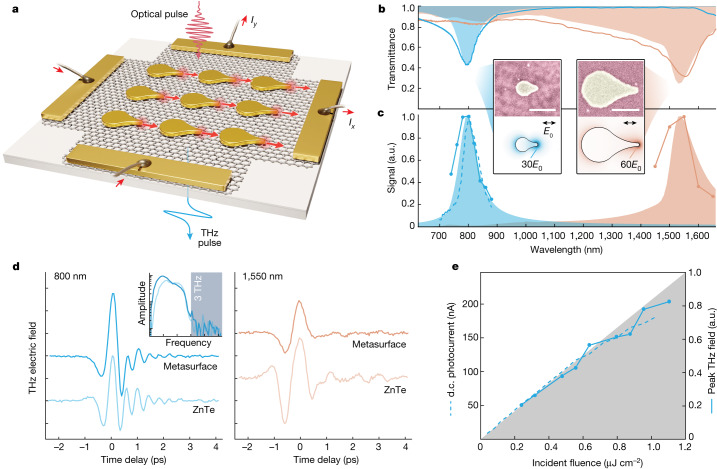
Directional photocurrents in symmetry-broken optoelectronic metasurfaces. **a**, Illustration of an optoelectronic metasurface consisting of symmetry-broken gold nanoantennas on graphene. Femtosecond laser illumination stimulates vectorial photocurrents and consequent emission of ultrafast THz pulses. **b**, Measured (solid line) and simulated (solid fill) transmission spectra for two nanoantenna designs with resonances at 800 nm (blue) and 1,550 nm (orange). **c**, Measured incident-wavelength-dependent THz field amplitude (solid lines with data markers) and d.c. photocurrent (dashed line), as well as simulated field intensity (solid fills). Top insets: scanning electron micrographs of the fabricated nanoantenna elements. Bottom insets: simulated plasmonic-field enhancements. Scale bars, 200 nm. **d**, Measured THz time-domain signals emitted from the resonantly excited 800-nm (left) and 1,550-nm (right) metasurfaces, compared with those from 1-mm-thick ZnTe. Curves are offset for clarity. Inset: amplitude spectra (plotted on a logarithmic scale) revealing bandwidth out to 3 THz, limited by phase matching in the 1-mm ZnTe detection crystal. **e**, Incident-fluence-dependent THz field amplitude and d.c. photocurrent readout, along with a linear fit to low fluence (solid fill). a.u., arbitrary units. Source Data

Our optoelectronic metasurface concept is illustrated in Fig. 1a, in which inversion-symmetry-broken gold nanoantennas act as lightning rods^29,30^, with strong, resonantly enhanced light fields at their sharp tips (approximately 15 nm radius; Fig. 1b,c, insets). Hot-carrier excitation at these nanoplasmonic hotspots drives local current density **j**(**r**, *t*) within the underlying graphene. The broken inversion symmetry leads to net current along the orientation of the nanoantennas, which decays on subpicosecond timescales owing to momentum-relaxing scattering with optical phonons, impurities and substrate phonons^31–33^. The transient photocurrents radiate free-space THz waves, **E**_THz_ ∝ −d**j**/d*t*, which are used as a contact-free probe of the ultrafast current dynamics. Furthermore, the time-averaged d.c. photocurrent is read out directly by means of electrical contacts for unambiguous verification of the overall charge-flow behaviours across millimetre-scale metasurfaces. These large-area devices offer larger currents and THz fields at relatively low fluences while also demonstrating the scalability and coherence of the subpicosecond photocurrents among millions of nanoscale directional sources, ultimately revealing the vectorial current behaviours at the nanoscale unit cell level.

We first investigate the photocurrent response in metasurfaces with uniform nanoantenna orientation and thus a globally preferred directionality. The plasmonic resonance is broadly tunable from visible to infrared wavelengths based on the nanoantenna structure (shape and size), material and dielectric environment. Structural tuning is exploited here for resonances at 800 nm and 1,550 nm, as clearly observed in the measured and simulated transmission spectra (Fig. 1b). Corresponding enhancements of the generated ultrafast THz fields and d.c. photocurrents are shown in Fig. 1c, in good agreement with the simulated optical-field-intensity enhancements, which underscores the central role of the (asymmetric) plasmonic fields in the photocurrent-generation process.

Single-cycle THz pulses are observed from both the 800-nm and 1,550-nm (Fig. 1d) metasurfaces through free-space electro-optic sampling (Methods), indicating the photocurrent rise and decay on timescales of a few hundred femtoseconds. These pulses disappear when the exposed graphene is etched away without removing the nanoantennas (Supplementary Note 1), verifying that the THz radiation originates from graphene photocurrents rather than optical rectification directly at the nanoantennas^34^. Remarkably, with little optimization (Supplementary Note 2), the 30-nm-thick metasurfaces yield THz fields that are comparable with or even stronger than those from widely used 1-mm-thick 〈110〉 ZnTe nonlinear crystals under the same excitation conditions. This indicates the potential use of these optoelectronic metasurfaces as new THz sources, with versatile responses arising from sample orientation and patterning, as well as incident polarization and wavelength, as explored further below.

A linear dependence on incident fluence (Fig. 1e) is observed in both the THz field amplitude and photocurrent readout for the 800-nm metasurface below about 0.8 µJ cm^−2^, which can result from various possible mechanisms at metal–graphene junctions, such as photovoltaic^15,35^ or photo-Dember^36^ effects. However, photothermoelectric effects are generally found to prevail at metal–graphene junctions, including under continuous-wave excitation at the nanoscale^26,27^ and ultrafast excitation at mesoscopic scales^37,38^. We will later show that photothermoelectric effects remain dominant under simultaneous ultrafast, nano-localized excitation, but with more intricate spatiotemporal energy and momentum flows. Before this, we explore the general implications of artificially patterned linear photocurrent responses, which can be extended to other material systems and are independent of the specific current-generation mechanism.

## Local vectorial photocurrent

**Fig. 2:**
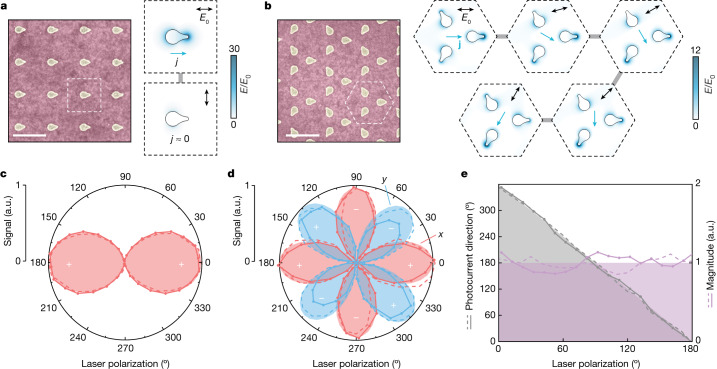
Polarization-dependent local responses and omnidirectional control. **a**,**b**, SEM images of uniformly oriented (**a**) and Kagome (**b**) metasurfaces with 800-nm resonances (overall metasurface size, 1 mm^2^). Scale bars, 500 nm. Insets: simulated resonant plasmonic-field enhancements for different incident linear polarization angles (black double arrows), with the calculated net current direction indicated (blue single arrows). **c**,**d**, Measured *x* (red) and *y* (blue) components of the radiated THz field (solid lines with data markers) and photocurrent (dashed lines) for the uniformly oriented (**c**) and Kagome (**d**) metasurfaces with respect to the incident linear polarization angle. Calculated linear responses (solid fills) are shown for comparison, with + and − signs indicating lobe polarity. For clarity, a small residual *y* component is not shown in **c**. **e**, The Kagome metasurface shows nearly constant photocurrent magnitude (purple) and continuously rotatable direction (grey), consistent with analytic predictions. Source Data

**Extended Data Fig. 1:**
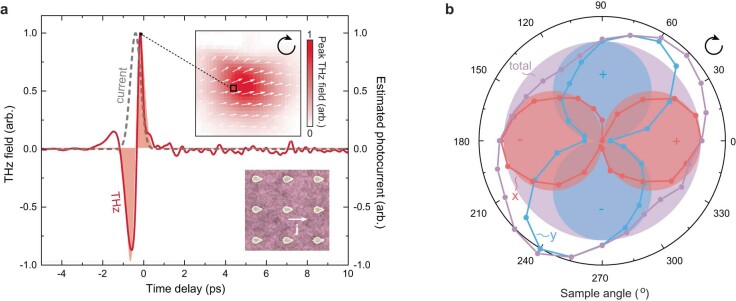
Linearly polarized THz emission from directional photocurrent. **a**, Measured (solid line) and approximated (solid fill) THz time-domain waveforms for a uniformly oriented metasurface under circularly polarized femtosecond laser excitation, assuming a Gaussian photocurrent pulse (dashed grey line; 600 fs full width at half maximum) with *E*_THz_ ∝ −d*j*/d*t*. The true current and THz field is expected to be faster than shown here, owing to bandwidth limitations of the photoconductive antenna detector (see Supplementary Note 1). Inset, spatially mapped THz field showing the beam with approximately uniform linear polarization. A small *y*-polarized contribution and corresponding roughly 10° angular deviation from *x* axis may be attributed in part to residual sample tilt and imperfect alignment of the THz imaging system. **b**, The *x* (red), *y* (blue) and total (purple) THz field amplitudes measured (solid lines with data markers) as a function of sample orientation under circularly polarized incident optical pulses, compared with the expected cos(*θ*) (red fill), sin(*θ*) (blue fill) and constant (purple fill) behaviours, respectively. The skew in the *y* dataset is attributed to imperfect circular polarization and residual misalignment in the parabolic mirrors. Source Data

For metasurfaces with uniform spatial patterning, as shown in Fig. 2a,b, global photocurrent and THz emission measurements reveal the local light–matter interaction symmetries and local vectorial (directionally tunable) photocurrent responses at the nanoscale unit cell level. The oriented metasurface (*pm* wallpaper group; Fig. 2a) exhibits a particularly simple cos^2^(*θ*) dependence on the incident linear polarization angle with respect to the nanoantenna principal axis of broken symmetry (Fig. 2c), which is characteristic of the linear dependence on the projected excitation-field intensity. The photocurrent directionality remains along this axis, regardless of the incident light polarization. Under circularly polarized incident light, the overall nanoantenna-oriented photocurrent across the metasurface is shown by the spatially uniform linear polarization of the radiated THz beam, which is continuously rotated by sample rotation (Extended Data Fig. 1).

Further symmetry can be introduced into the metasurface unit cell with several nanoantennas oriented in different directions. In general, a unit cell with *C*_*n*_ rotational symmetry will exhibit an isotropic linear response if *n* > 2, with noncentrosymmetric configurations (odd *n*) required for net directional photocurrents. We apply this general principle with a Kagome lattice metasurface (Fig. 2b), which has threefold rotational symmetry and three planes of mirror symmetry (*p*3*m*1 wallpaper group). Global and corresponding local photocurrent directionality is continuously rotated by varying the incident linear polarization angle (Fig. 2d,e), with a linear combination of hotspot excitations (Fig. 2b) maintaining a constant photocurrent magnitude. This omnidirectional local vectorial current control is described in a Cartesian basis by ${j}_{x}=j{\sum }_{i}{\cos }^{2}(\theta -{\theta }_{i})\cos \left({\theta }_{i}\right)$ and ${j}_{y}=j{\sum }_{i}{\cos }^{2}(\theta -{\theta }_{i})\sin \left({\theta }_{i}\right)$, in which *θ* is the laser polarization angle and *θ*_*i*_ = 0, 2π/3 and 4π/3 for the three nanoantenna orientations within the Kagome unit cell.

**Extended Data Fig. 2:**
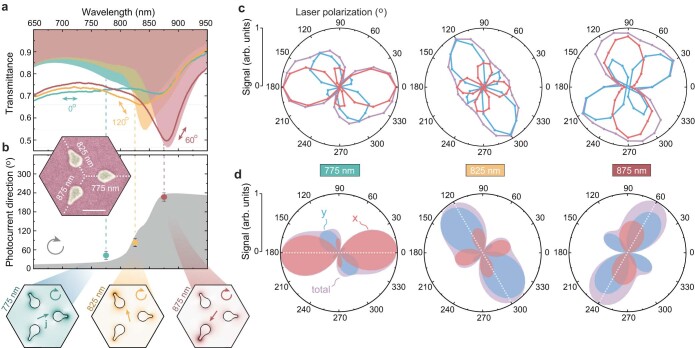
Wavelength-sensitive directional photocurrent response. **a**, Measured (solid lines) and simulated (solid fills) transmittance spectra for the perturbed Kagome lattice, with white light linearly polarized along the three nanoantennas and resonances nominally designed around 775 nm (0°; cyan), 825 nm (120°; orange) and 875 nm (240°; red). Inset, unit cell (scale bar, 250 nm). **b**, Direction of THz field polarization (and thus net photocurrent) measured (solid circles) and simulated (solid fills) as a function of incident wavelength for circularly polarized incident light. Bottom insets show simulated fields and net current directions for circularly polarized excitation at the three resonant wavelengths. Measured (**c**) and simulated (**d**) *x* (red), *y* (blue) and total (purple) THz fields as a function of incident linear polarization angle at the three resonance wavelengths, showing preferred alignment along the active resonance axes (dotted white lines). Source Data

These prototypical metasurfaces already illustrate a broad design space for nanoscale vectorial photocurrents, which can be driven in arbitrary directions in space and/or time under different incident light fields. Localized vortical charge flows with no net global current can be realized under circularly polarized excitation, for instance, by rotating the nanoantenna orientations within the Kagome metasurface unit cell (Supplementary Fig. 11). The design space grows further with the introduction of different structures and resonant wavelengths. In such systems, the light–matter interaction symmetry may even be different from the underlying structural symmetry and tunable with wavelength, as read out by the polarization-dependent and wavelength-dependent photocurrent response. Such possibilities are demonstrated in Extended Data Fig. 2, in which the Kagome metasurface unit cell contains three resonators of different dimensions. Although this perturbed Kagome lattice evidently exhibits the lowest possible degree of symmetry (*p*1 wallpaper group), an approximate mirror symmetry in the light–matter interaction appears either along a dominant resonance axis or between two equally excited resonances at intermediate wavelengths^25^. The general linear photocurrent response for any metasurface with uniform spatial patterning is given by ${j}_{x}(\theta ,\lambda )\propto {\sum }_{i}{E}_{i}^{2}(\lambda ){\cos }^{2}(\theta -{\theta }_{i})\cos \left({\theta }_{i}\right)$ and ${j}_{y}(\theta ,\lambda )\propto {\sum }_{i}{E}_{i}^{2}(\lambda ){\cos }^{2}(\theta -{\theta }_{i})\sin \left({\theta }_{i}\right)$, in which *E*_*i*_(*λ*) are the wavelength-dependent plasmonic fields at each tip hotspot. This shows a high degree of adaptability, with responses that are not rigidly fixed by metasurface patterning. These results also directly demonstrate important capabilities for bias-free polarization-resolved (Fig. 2d,e) and wavelength-resolved (Extended Data Fig. 2) photodetection for integrated, ultrafast optoelectronic and information science applications^39^.

## Spatially varying vectorial photocurrent

**Fig. 3:**
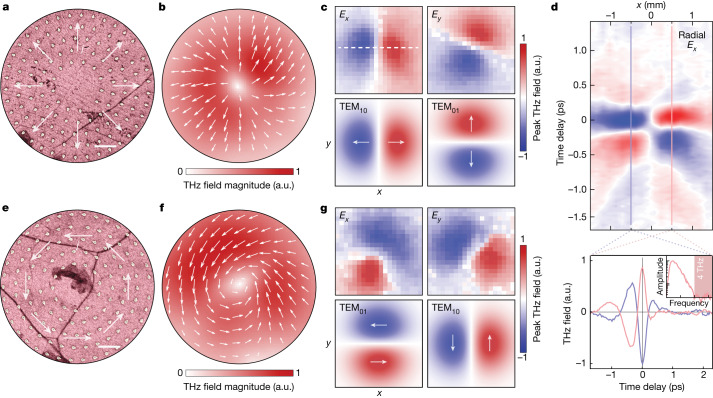
Global vectorial currents and THz vector beams. **a**, SEM image of the central region of a radial vector metasurface, with arrows illustrating the expected radial photocurrent on circularly polarized excitation. The entire metasurface (1 mm overall diameter) is excited. Scale bar, 1 µm. **b**, Far-field spatial map of the radial THz vector field, showing the total measured THz field magnitude (colour map and length of white arrows) and direction (direction of white arrows) at the pulse peak. **c**, Measured (top) versus ideal (bottom) Hermite–Gaussian modes for the *x* and *y* field components of the radial THz vector beam. Arrows in bottom plots indicate THz field polarity at peak time. **d**, Lineout from the radial *E*_*x*_ image, showing the transient THz field as a function of *x* position, along with example THz time-domain waveforms illustrating the polarity reversal across the beam. Inset: corresponding THz field amplitude spectrum in the frequency domain, plotted on a logarithmic scale. **e**–**g**, The same as in **a**–**c** but for the azimuthal vector metasurface and THz vector beam. Source Data

**Extended Data Fig. 3:**
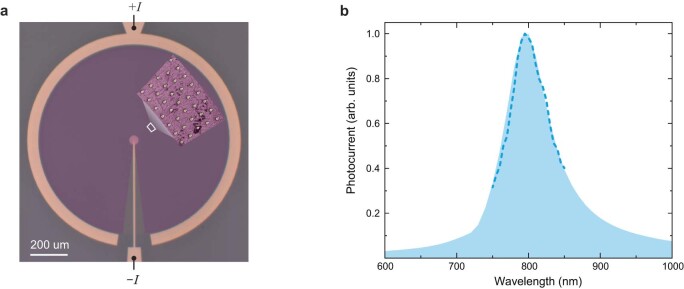
Radial metasurface device and resonant excitation. **a**, Optical micrograph of a radial metasurface, prepared as a device for photocurrent readout. Inset, radially outward-oriented nanoantennas with resonance at 800 nm and nanoantenna spacing about 500 nm. **b**, Measured DC photocurrent (dashed line) and simulated plasmonic hotspot field intensity (solid fill; same as in Fig. 1c), verifying the resonantly coupled/enhanced radial photocurrent. Source Data

Nonuniform spatial patterning can be implemented for nearly arbitrary control over spatially varying vectorial current distributions out to macroscopic scales, as demonstrated with radial (Fig. 3a) and azimuthal (Fig. 3e) metasurfaces. The polarization patterns of the radiated THz beams reveal the global radial and azimuthal transient charge flows generated on circularly polarized excitation across the entire 1-mm metasurfaces. We thus use a high-throughput THz emission spectroscopy system for hyperspectral imaging (Fig. 3d), scanning across the far-field THz beam and reading out the full time, frequency and polarization information as a function of position (Methods). These maps show clear radially polarized (Fig. 3b,c) and azimuthally polarized (Fig. 3f,g) THz fields, unambiguously confirming the spatially varying vectorial photocurrents. For the radial metasurface device, the radial current and resonantly enhanced excitation are also verified by direct photocurrent readout measurements (Extended Data Fig. 3).

Although the mapped THz fields serve to demonstrate the spatially varying vectorial currents, they also illustrate a new capability for direct generation of broadband vector beams in the THz frequency range. The inset to Fig. 3d indicates spectral amplitude above the noise floor out to 4 THz, but comparison with a broadband spintronic emitter shows this to be limited by the spectral response of the photoconductive antenna detector (Supplementary Fig. 3). These cylindrical THz vector beams hold substantial promise for THz imaging, spectroscopy and other applications^17^. Although various schemes have emerged recently for generating THz vector beams^17^, they often rely on bulky, narrowband and/or multistage systems. By contrast, direct generation of arbitrary THz vector fields from spatially patterned photocurrents offers an ultrathin, broadband and single-stage source that can also be actively manipulated by incident optical fields or by electrostatic gating, as examined next.

## Photothermoelectric dynamics

**Fig. 4:**
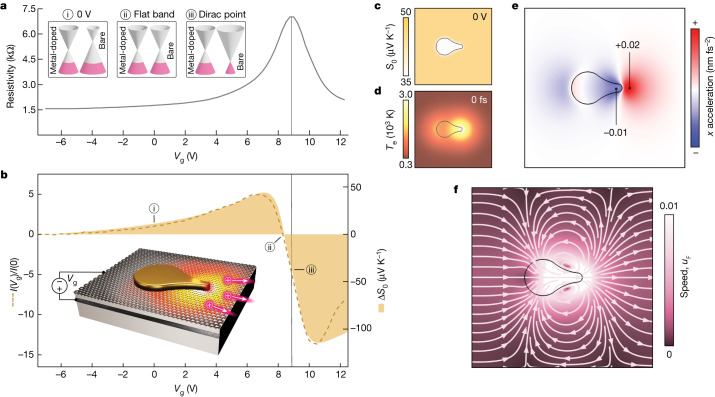
Local photothermoelectric driving force and resulting nanoscale charge flow. **a**, Measured resistivity of a metasurface device as a function of gate voltage. Insets: chemical potentials of the gold-pinned and bare graphene regions for three gate voltages as indicated in **b**. **b**, Measured gate-dependent current (dashed line) compared with calculated Δ*S*_0_ (solid fill). Inset: illustration of the gated device configuration, with tip-localized heating and corresponding net carrier motion. **c**, Spatial distribution of *S*_0_. **d**, Snapshot of the calculated *T*_e_ distribution at the time of the incident pulse peak (0 fs). **e**, Photothermoelectric *x* acceleration field (along the principal nanoantenna axis) at *V*_g_ = 0 V and 0 fs. **f**, Hydrodynamic velocity field for the hole fluid at *V*_g_ = 0 V and 0 fs. Source Data

We further investigate the dynamics that occur within the gold–graphene metasurfaces under femtosecond optical excitation at the nanometric-tip hotspots. The photocurrent driving mechanism is revealed by electrostatic gating of large-area metasurface devices (Methods), with a back-gate voltage *V*_g_ applied to tune chemical potential *µ* and corresponding electrical conductivity *σ* in the bare graphene regions (Fig. 4a). By contrast, the chemical potential of the graphene beneath the nanoantennas remains pinned to that of gold^40^, leading to a spatially varying Seebeck coefficient (Fig. 4c) described by the Mott relation at room temperature *T*_0_, ${S}_{0}=-\frac{{{\rm{\pi }}}^{2}{k}_{{\rm{B}}}^{2}{T}_{0}}{3e}\frac{1}{\sigma }\frac{{\rm{d}}\sigma }{{\rm{d}}\mu }$, in which *k*_B_ is the Boltzmann constant, *e* is the elementary charge and $\left|\mu \right|\propto {\left|{V}_{{\rm{g}}}-{V}_{{\rm{CNP}}}\right|}^{1/2}$ with *V*_CNP_ ≈ 9 V at the charge-neutrality point (Supplementary Note 1). The photocurrent closely follows the nonmonotonic dependence of the difference Δ*S*_0_ between the bare and gold-doped graphene regions (Fig. 4b), which is a clear signature of a photothermoelectric effect^41,42^ and is inconsistent with photovoltaic (monotonic *V*_g_ dependence) or other linear responses that can occur at graphene junctions^15,35,36^.

The dynamics studied here are extremely confined in both space and time compared with previous investigations under continuous-wave excitation or at mesoscopic junctions^26,37,38,41–44^. We therefore combine electromagnetic, thermodynamic and hydrodynamic modelling to understand the evolution of the far-from-equilibrium electronic temperature *T*_e_(**r**, *t*), the effective photothermoelectric driving field ∝ −*S*(**r**, *t*)∇*T*_e_ and the resulting nanoscale charge flow **j**(**r**, *t*) in graphene. The localized optical power absorption generates a peak *T*_e_ approaching 3,000 K around the nanoantenna tip (Fig. 4d) for an incident pulse fluence of 0.5 µJ cm^−2^ in the linear response regime. In-plane electronic thermal diffusion and scattering with optical phonons^31^ along with out-of-plane scattering with substrate phonons^32^ then cool the superheated carrier distribution within a few hundred femtoseconds (Supplementary Note 3). Before cooling, a net acceleration proportional to Δ*S*∇*T*_e_ acts on the electronic system along the nanoantenna axis (Fig. 4e). We note that, although Δ*S* exhibits a nonlinear temperature dependence at high *T*_e_ (Supplementary Note 3), this is not expected to alter the gating behaviour shown for Δ*S*_0_ (Fig. 4b) beyond a scaling factor. The resulting charge flow is modelled by means of the time-dependent incompressible Navier–Stokes equation (Fig. 4f and Methods). Such a hydrodynamic framework^45–47^ is based on the condition that *τ*_ee_ ≪ *τ*_mr_, in which *τ*_ee_ is the electron–electron scattering time and *τ*_mr_ is momentum relaxation time associated with electron impurity/phonon scattering. This treatment can be justified even for the relatively low-mobility graphene (*τ*_mr_ ≈ 45 fs) by the electronic superheating, which markedly increases the phase space for electron–electron scattering. On photoexcitation, ${\tau }_{{\rm{ee}}}\propto {T}_{{\rm{e}}}^{-2}$ is driven from a few hundred femtoseconds at room temperature into the few-femtoseconds range at several thousand kelvin (Supplementary Note 3), supporting a local hydrodynamic treatment and the possibility of light-induced viscous electron flow^48^.

**Extended Data Fig. 4:**
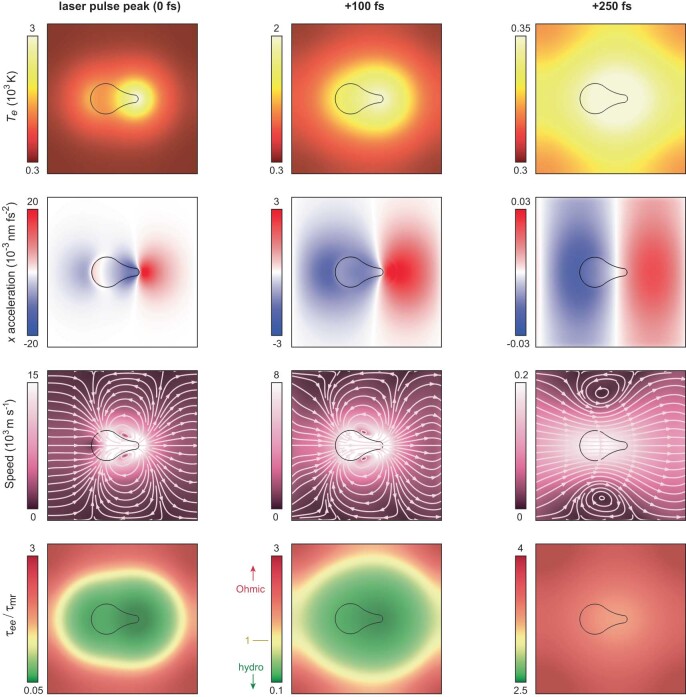
Ultrafast time evolution of local thermodynamic and hydrodynamic quantities. Calculated electron temperature, photothermoelectric acceleration field, hydrodynamic flow profile and relative scattering rates within the square metasurface unit cell as a function of time with respect to the 100-fs laser pulse. All plots are normalized to the peak values.

Indeed, this multiphysics modelling shows the rapid evolution of *T*_e_, *τ*_ee_ and corresponding local physical quantities (for example, electron viscosity, thermopower and heat capacity) that occurs over timescales of hundreds of femtoseconds and spatial scales of tens of nanometres (Extended Data Fig. 4). These calculations provide insight on the local nanoscale dynamics while also capturing the resulting global behaviours measured for the various metasurfaces, including polarization, frequency and intensity dependencies (Supplementary Note 4), predicting time-averaged photocurrents that agree to within a factor of 2 of experimental measurements (Supplementary Fig. 10).

## Discussion

The measurements and modelling discussed here explain the ultrafast nanoscale charge flows that yield net local vectorial and spatially varying vectorial photocurrents. Combining structure-based patterned symmetries and light-based control in this way opens up new domains for driving otherwise unrealizable charge currents in materials. Beyond gold and graphene, these effects can be explored in an expansive selection of systems, with photocurrent responses expected owing to photothermoelectric, photovoltaic and photoinjection^49^ effects in semiconductors, semimetals, topological insulators, ferromagnets and other materials. Symmetry-broken nanoscale structuring may also be imposed on new materials to examine unknown local responses and physical properties. Furthermore, introduction of planar chirality allows for dynamic control over nanomagnetic moments (Supplementary Fig. 12). More generally, spatially varying photocurrents can be used to generate complex magnetic fields^50^ in free space or nearby materials. Although here we have directly demonstrated polarization/wavelength-resolved photodetection and ultrafast THz generation in symmetry-broken optoelectronic metasurfaces towards information science, microelectronics and THz technologies, we anticipate that many more opportunities will emerge in other applications (for example, nanomagnetism, materials diagnostics, ultrafast information processing and energy harvesting) using the basic concepts introduced in this work.

## Methods

### Metasurface fabrication

Metasurface fabrication begins with large-area monolayer graphene grown by chemical vapour deposition and transferred onto fused quartz substrates (Supplementary Note 1). Substrates are spin-coated with bilayer poly(methyl methacrylate) (PMMA) (PMMA 495 bottom layer and PMMA 950 top layer) at 3,000 rpm for 30 s, followed by 1 min prebake at 180 °C. A conductive polymer layer (DisCharge) is then applied to ensure adequate charge dissipation during electron-beam exposure. Arrays are then patterned through electron-beam lithography using a 100-kV JEOL JBX-6300FS system. For <30 nm features, interparticle proximity effect correction is used with an optimized total dosage around 1,650 µC cm^−2^. Under these conditions, writing of a 1-mm^2^ array takes approximately 1 h. Following a 30-s rinse in isopropanol for DisCharge removal, samples are then developed in a 3:1 isopropanol:methyl isobutyl ketone solution for 1 min. A 30-nm gold layer is then deposited by means of electron-beam or sputter deposition without any adhesion layer. Liftoff is performed after overnight acetone soaking, using gentle acetone wash bottle rinsing to remove the residual PMMA/gold. For most samples, no particle delamination is observed.

Devices for simultaneous ultrafast THz emission and average photocurrent electrical readout studies are prepared using maskless photolithography (Heidelberg MLA 150). We use AZ 5214 E photoresist in image-reversal mode, precoating the samples with a hexamethyldisilazane layer to promote photoresist adhesion. Excess graphene is etched to obtain desired geometries by means of 2-min exposure to O_2_ plasma (100 W, 10 sccm; Anatech RIE). Electrodes consist of 50-nm Au with a 3-nm Ti adhesion layer.

For back-gated devices, the back-gate electrode with minimal overlap with the top electrodes (precluding any shorting through defects in the dielectric spacer layer) is prepared through photolithographic patterning, deposition of 3 nm/30 nm Ti/Pt and a liftoff process. Various spacer-layer materials (SiO_2_, Al_2_O_3_ and HfO_2_), deposition methods (atomic layer deposition and physical vapour deposition) and thicknesses (5–100 nm) are tested for optimal electrostatic gating of large-area devices. We find that 30-nm SiO_2_ offers a good trade-off between high gating capacitance and relatively modest nanoantenna resonance redshifting owing to the image charge oscillation within the Pt and the dielectric environment of the spacer layer. Subsequent device preparation follows as described above.

### Basic characterization

The quality of metasurface fabrication is characterized by means of optical microscopy, scanning electron microscopy (SEM) and white-light transmission spectroscopy. Optical bright-field and dark-field imaging under 50× magnification is sufficient for resolving individual nanostructures to verify successful liftoff and large-scale sample uniformity while also resolving the monolayer graphene edge in etched devices. A more detailed view of the graphene and nanostructure morphologies (Supplementary Figs. 1 and 2) is provided by means of SEM micrographs collected using FEI Magellan, Nova NanoSEM 450 and Nova NanoLab 600 systems. The fabricated nanoantennas closely reproduce the design profile down to the tens-of-nanometres scale, with extra-sharp approximately 15 nm radius of curvature tips.

Metasurface optical properties are verified by means of white-light transmission spectroscopy using a tungsten-halogen white-light source passed through a broadband polarizer for linear polarization control. Two 20× microscope objectives are used to focus the light through the sample and collect the transmitted light into both visible (Acton SP2300) and near-infrared (Ocean Optics NIRQuest) spectrometers. The transmittance is given by the ratio of signal (on the metasurface) to reference (off the metasurface but on the same substrate), with the ambient background collected with the white-light source turned off and subtracted from each.

### THz emission spectroscopy

THz emission experiments are performed across several systems with different functionality, with overlapping datasets verifying reproducibility. All measurements except wavelength-dependent THz emission (see below) are performed in the low-fluence regime (<1 µJ cm^−2^) using a Ti:sapphire oscillator (Chameleon Vision-S) with 800-nm, roughly 100-fs pulses at the sample location at 80 MHz repetition rate. As seen in Fig. 1e, these low fluences ensure linear responses and also preclude thermally induced nanoantenna deformation or photochemical degradative effects^51^ that can occur in the intense hotspot regions. The gradual onset of a sublinear regime beyond about 0.8 µJ cm^−2^ in Fig. 1e is attributed to competing temperature-dependent thermodynamic contributions (Supplementary Notes 3 and 4). For metasurfaces operating at 800 nm, the THz radiation is measured through electro-optic sampling^52^ using a 1-mm 〈110〉 ZnTe crystal. Three wire-grid polarizers are used to measure the *x* and *y* field components of the THz radiation, with the first polarizer at 0° or 90°, the second polarizer at 45° and the third polarizer at 0°. The THz beam path is contained within a dry-air-purged environment (<2% relative humidity).

Pump-wavelength-dependent THz emission experiments are performed using a noncollinear optical parametric amplifier to generate pump pulses across 725–875 nm (pulse width ≤ 50 fs) and an optical parametric amplifier to generate pump pulses across 1,450–1,650 nm (pulse width ≤ 200 fs). Both amplifiers are seeded by a 1-MHz fibre-amplified solid-state laser producing approximately 270-fs pulses at 1,032 nm. In all of these measurements, we tune the pump wavelength while the incident fluence is maintained at a constant value using a neutral-density attenuator. The direct output of the seed laser is used to gate the electro-optic sampling with a 0.5-mm ⟨110⟩ GaP crystal. This enables wavelength-dependent pumping without affecting the detection.

For spatial mapping of THz vector beams, we use a custom Menlo Systems apparatus consisting of a 100-MHz mode-locked 1,560-nm erbium-doped all-fibre laser oscillator that seeds two separate amplifiers. The first line uses a high-power erbium fibre amplifier driven in the linear pulse propagation regime, followed by free-space second-harmonic generation, outputting 1 W of 780-nm, 125-fs pulses and serving as the pump beam for generation of THz radiation from metasurfaces. The second amplifier line is used to gate a broadband, fibre-coupled photoconductive antenna detector^53^ (TERA 15-RX-FC), which is mounted on a 2D stage for automated *xy* spatial scanning. Along with the high-repetition integrated delay stage (>45 Hz for a 20-ps temporal scanning range), this system enables rapid hyperspectral imaging of THz vector beams, which are collimated and refocused by two TPX lenses (50 mm focal length). The photoconductive antenna is situated before the focal point of the second lens, with resulting deviations from a planar phase front that are calibrated and corrected for in the results presented in Fig. 3.

### Device gating and photocurrent readout

**Extended Data Fig. 5:**
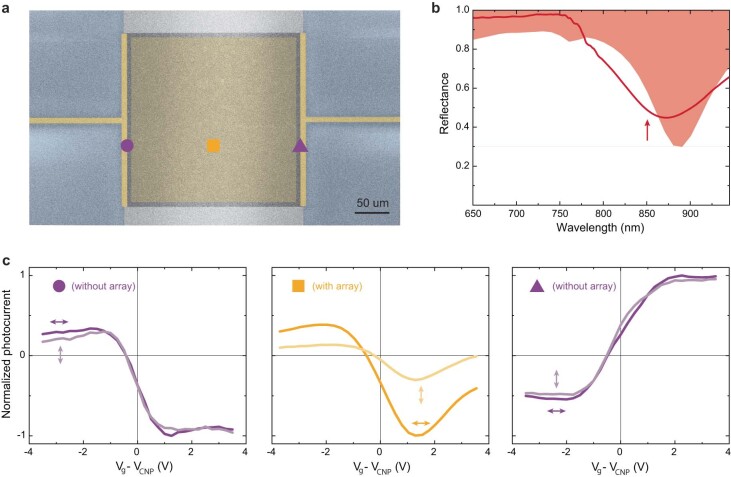
Electrostatically gated metasurface. **a**, Metasurface device, with false colouring indicating the 3-nm/50-nm Ti/Au (light yellow), nanoantenna array (darker yellow), graphene (dark grey), 30-nm Pt back-gate electrode with 30-nm silica spacer layer (light grey) and substrate (light blue). **b**, Measured (solid line) and simulated (solid fill) reflectance spectrum of the device, showing resonant absorption around 875 nm. **c**, Gate-dependent photocurrent as a function of position, with locations indicated in **a**. Beam spot about 25 µm. To isolate the electrode contributions, the left and right electrode measurements are performed on an array-free device, with little laser polarization dependence observed. A polarity reversal is observed between the left and right electrodes. By contrast, strong polarization dependence is observed on the array at the centre of the sample. Both metasurface and array-free devices have 250-µm square graphene between the electrodes (metasurface device is the same as in Fig. 4). Photocurrents are normalized to the peak positive or negative values. Source Data

Time-averaged photocurrents are electrically read out using a lock-in amplifier (for the highest signal-to-noise ratio) and picoammeter (to directly measure polarity). Gating studies are performed using a computer-controlled dual-channel sourcemeter (Keithley 2614B) for simultaneous application of a gate voltage and readout of either the device resistance (using a sourcemeter) or photocurrent (using a picoammeter or lock-in amplifier). Although nonlocal photocurrents in graphene devices are often evaluated in terms of a Shockley–Ramo-type response^1,54^, the square metasurface and electrode geometries here lead to a simplified, direct readout of the overall *x* and *y* photocurrent components. Wavelength-dependent photocurrent responses are measured by directly tuning the output wavelength of the 80-MHz Ti:sapphire laser while maintaining a constant power. Although the electrode gold–graphene junctions can also contribute to the photothermoelectric current, the current induced by the nanoantennas is isolated through: (1) the spatial position of the focused beam; (2) the much stronger nanoantenna polarization dependence; and (3) the lack of a similar response in nanoantenna-free devices (see Extended Data Fig. 5 for further details).

### Dynamical multiphysics model

Coupled electromagnetic, thermodynamic and hydrodynamic equations are solved using the finite element method (COMSOL Multiphysics 6.1). Nanoscale optical fields are first simulated using a 3D rectangular metasurface unit cell domain consisting of the air superstrate, gold nanoantennas (dielectric function given by Johnson and Christy^55^), conductive graphene boundary layer and fused quartz substrate^56^. Periodic (Floquet) boundary conditions are applied in the transverse *x* and *y* directions, with input/output ports along the *z* direction backed by perfectly matched layers. The graphene optical conductivity is approximately constant in the near-infrared frequency range^57^, with real component *σ*_r_ = 6.1 × 10^−5^ Ω^−1^ and imaginary component *σ*_i_ = −2.1 × 10^−5^ Ω^−1^. Resonant transmission spectra are obtained from the simulated S-parameters under plane-wave excitation at normal incidence, consistent with the experiments. The same nanoantenna geometry file is used for both lithography and simulations.

The full thermal evolution of the 2D graphene electron and lattice (specifically, optical phonon^31^) systems is calculated through the coupled heat equations

1a $${c}_{{\rm{e}}}\frac{\partial {T}_{{\rm{e}}}}{\partial t}=\frac{1}{2}{\sigma }_{{\rm{r}}}{E}^{2}+\nabla \cdot ({\kappa }_{{\rm{e}}}\nabla {T}_{{\rm{e}}})-{g}_{{\rm{er}}}({T}_{{\rm{e}}}-{T}_{{\rm{op}}})-{g}_{{\rm{sub}}}({T}_{{\rm{e}}}-{T}_{0}),$$

1b $${c}_{{\rm{op}}}\frac{\partial {T}_{{\rm{op}}}}{\partial t}={\kappa }_{{\rm{l}}}{\nabla }^{2}{T}_{{\rm{op}}}+{g}_{{\rm{er}}}({T}_{{\rm{e}}}-{T}_{{\rm{op}}}),$$

in which *c*_e_ is the volumetric electronic specific heat described in Supplementary Note 3, ${\kappa }_{{\rm{e}}}=\frac{1}{3}{\left(\frac{{\rm{\pi }}{k}_{{\rm{B}}}}{e}\right)}^{2}{T}_{{\rm{e}}}\sigma$ is the in-plane electronic thermal conductivity approximating Wiedemann–Franz law behaviour (approximately preserved in the Fermi liquid regime of graphene^58^), *g*_sub_ = 3 MW m^−2^ K^−1^ is used as the out-of-plane electronic thermal conductance owing to coupling with the SiO_2_ substrate phonons^32^ (corresponding to a cooling length $\sqrt{{\kappa }_{{\rm{e}}}/{g}_{{\rm{sub}}}}\approx 150\,{\rm{nm}}$ at 3,000 K), *T*_op_ is the optical phonon temperature, *c*_op_ is the optical phonon specific heat based on time-resolved Raman studies of graphite^31,59^ and *κ*_l_ ≈ 1.7 × 10^−7^ W K^−1^ is the lattice thermal conductance based on thermal transport measurements of supported graphene^60^ (1:1 device aspect ratio here). The energy relaxation electron–optical phonon coupling constant, *g*_er_ ≈ 2 × 10^7^ W K^−1^ m^−2^, is estimated from transient reflectivity measurements (Supplementary Note 3). The source term, $\frac{1}{2}{\sigma }_{{\rm{r}}}{E}^{2}$, is the absorbed power density from the transient laser pulse field $E(t)={E}_{0}{{\rm{e}}}^{-2{\rm{ln}}2{t}^{2}/{\tau }_{{\rm{p}}}^{2}}$, in which *E*_0_ is the peak field determined in the frequency-domain electromagnetic simulations and *τ*_p_ = 100 fs is the laser pulse duration. Coupling with the acoustic phonon bath on longer picosecond timescales and radiative loss are neglected. The effect of Peltier cooling—which would appear as $-{T}_{{\rm{e}}}\nabla S\cdot {\bf{j}}$ on the right-hand side of the heat equation—is found to be small. Further details on the thermodynamic modelling are provided in Supplementary Note 3, including the thermodynamic quantities in the high-*T*_e_ limit in graphene and simplified two-temperature models for the electron and lattice temperature evolution.

The electronic temperature evolution (energy flow) described by equation (1) drives momentum flow by means of photothermoelectric force **f**_PTE_ ∝ ∇*T*_e_, as modelled by the time-dependent linearized Navier–Stokes equation

2 $$\frac{\partial {\bf{u}}}{\partial t}-\nabla \cdot (\nu \nabla {\bf{u}})+\frac{1}{{\tau }_{{\rm{mr}}}}{\bf{u}}={{\bf{f}}}_{{\rm{PTE}}},$$

assuming incompressible flow of the charged fluid

$$\nabla \cdot {\bf{u}}=0.$$

Here **u** is the 2D electron velocity field (current density **j** = *en*_e_**u** with *n*_e_ the charge density) and *ν* is the temperature-dependent kinematic electron viscosity. As is typical for electron hydrodynamic flow, the Reynolds number is small and the contribution of the nonlinear convective term $({\bf{u}}\cdot \nabla {\bf{u}})$ is found to be negligible. The electron kinematic viscosity depends linearly on the electron–electron scattering time, *ν* ∝ *τ*_ee_, which therefore scales locally as approximately ${T}_{{\rm{e}}}^{-2}$ (Supplementary Note 3). The diffusive term is written as $\nabla \cdot (\nu \nabla {\bf{u}})$, similar to the diffusive term in the heat equation, to properly account for the spatial variation of the kinematic viscosity (Extended Data Fig. 4). The temperature dependence of *S* in the force term (**f**_PTE_ = −*qS*∇*T*_e_, in which *q* = ±*e*) is determined by solving the generalized Mott relation (Supplementary Note 3). Small contributions from Joule heating, which depend quadratically on the current density, are not included in the present treatment. The driving force **f**_PTE_ is highly nonuniform in space and, consequently, the spatial profile of the velocity flow is not simply governed by the length scale determined from the momentum relaxation and viscosity or the Gurzhi length, $\sqrt{\nu {\tau }_{{\rm{mr}}}}$. The resulting ultrafast, nanoscale spatiotemporal evolution of the electronic temperature, force field, charge flow and *τ*_*ee*_/*τ*_mr_ are shown in Extended Data Fig. 4, with further discussion of the hydrodynamic modelling provided in Supplementary Note 4.

## Online content

Any methods, additional references, Nature Portfolio reporting summaries, source data, extended data, supplementary information, acknowledgements, peer review information; details of author contributions and competing interests; and statements of data and code availability are available at 10.1038/s41586-024-07037-4.

### Supplementary information

Supplementary Information This file contains Supplementary Figs. 1–11, Notes 1–4 and References.

### Source data

Source Data Fig. 1

Source Data Fig. 2

Source Data Fig. 3

Source Data Fig. 4

Source Data Extended Data Fig. 1

Source Data Extended Data Fig. 2

Source Data Extended Data Fig. 3

Source Data Extended Data Fig. 5

## Data Availability

All data presented in this work are fully available from the corresponding authors. Source data are provided with this paper.

## Code availability

All calculations and simulations in this work are performed with standard packages, with all relevant information for reproduction described in the text and supplementary materials. COMSOL and other calculation files are available from the corresponding authors on request.
