# Supplementary material for: Light-driven nanoscale vectorial currents
Source: Nature. 2024 Feb 7;626(8001):984–9. doi: 10.1038/s41586-024-07037-4 (PMC10901733; doi:10.1038/s41586-024-07037-4)
Supplement: Supplementary file 1 — This file contains Supplementary Figs. 1–11, Notes 1–4 and References. [file 41586_2024_7037_MOESM1_ESM.pdf]

nature portfolio

https://doi.org/10.1038/s41586-024-07037-4

Supplementary information

Light-driven nanoscale vectorial currents

In the format provided by the authors and unedited

Supplementary Information for

Light-Driven Nanoscale Vectorial Currents

Jacob Pettine[1]*, Prashant Padmanabhan[1], Teng Shi[1], Lauren Gingras[2], Luke McClintock[1,3], Chun-Chieh Chang[1], Kevin W. C. Kwock[1,4], Long Yuan[1], Yue Huang[1], John Nogan[5], Jon K. Baldwin[1], Peter Adel[2], Ronald Holzwarth[2], Abul K. Azad[1], Filip Ronning[6], Antoinette J. Taylor[1], Rohit P. Prasankumar[1,7], Shi-Zeng Lin[1], and Hou-Tong Chen[1]*

[1] Center for Integrated Nanotechnologies, Los Alamos National Laboratory, Los Alamos, NM 87545, United States

[2] Menlo Systems, Martinsried, Bavaria 82152, Germany

[3] Department of Physics, University of California-Davis, Davis, CA 95616, United States

[4] The Fu Foundation School of Engineering and Applied Science, Columbia University, New York, NY 10027, United States

[5] Center for Integrated Nanotechnologies, Sandia National Laboratories, Albuquerque, NM 87123, United States

[6] Institute for Material Science, Los Alamos National Laboratory, Los Alamos, NM 87545, United States

[7] Intellectual Ventures, Bellevue, WA 98005, United States

* jacob.pettine@lanl.gov, chenht@lanl.gov

Contents

Supplementary Figures S1–S11

Supplementary Note 1: Sample characterization 2

Supplementary Note 2: Electromagnetic response 5

Supplementary Note 3: Thermodynamics 7

Supplementary Note 4: Hydrodynamic flow 14

Supplementary References 17

Supplementary Note 1: Sample characterization

1.1 Monolayer graphene properties

Large-area (1 cm^2) graphene monolayers are obtained from commercial sources (Graphenea and ACS Material), with similar properties from both sources, grown via chemical vapor deposition (CVD) and transferred onto fused quartz substrates or gating devices by the standard wet transfer method using polymethyl methacrylate (PMMA)[1]. Water molecules trapped within the graphene/substrate interface during this process enhance the p-type graphene doping and can contribute to hysteresis under applied back-gate voltages (V_g) during electrostatic gating[2]. While hysteresis is observed within our electrostatic gating measurements, making the precise determination of the chemical potential more challenging, the photocurrents follow the measured resistivities in a consistent manner. We therefore utilize only the forward V_g scans and corresponding photocurrents to gain physical insight on the photothermoelectric process.

Common microscopic domain structure for CVD graphene, including wrinkles and multilayer nucleation sites[1], are observed in scanning electron micrographs (Fig. S1). Although these defects contribute to momentum-relaxing scattering events and thereby limit global mobilities, little influence on the metasurface response is expected due to their small filling factor and random distribution at mesoscopic scales. For radial and azimuthal metasurfaces, for instance, graphene imperfections evident in the electron micrographs of the central array regions (Fig. 3a,e) are randomly distributed across the 1 mm-scale metasurfaces and we find no observable change in the THz vector fields upon sample rotation, indicating the minimal role of any metasurface defects on the global currents. Furthermore, only a small difference in the terahertz (THz) emission strength is observed for 1–4-layer samples, discussed below (Fig. S5). Carrier mobilities of $\mu_e \approx$ 1500 $cm^2V^{-1}s^{-1}$ are determined by the resistivity of large-area ($\geq 200 \times 200$ μm^2) nano-patterned devices with negligible contact resistance. This is in good agreement with Hall mobilities measured on smaller Hall bar devices without nanostructures, indicating that residual resist and other factors from the lithography steps do not contribute significantly to scattering. Given these

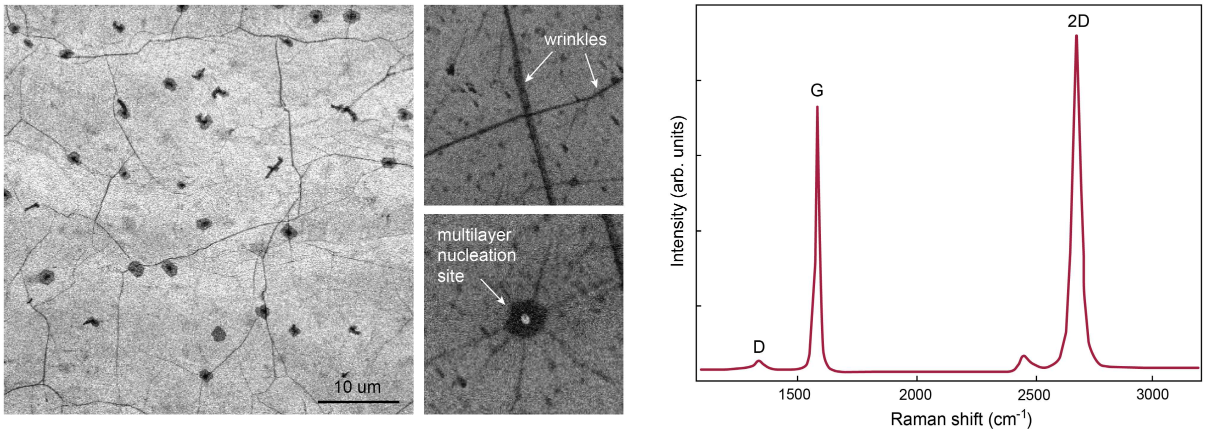

Fig. S1 | Graphene properties. (Left) Scanning electron micrographs showing typical features of large-area CVD graphene, including wrinkles and bilayer/multilayer nucleation sites from which the monolayer domains grow. (Right) Raman spectrum of the monolayer graphene, with $I_{2D} > I_G \gg I_D$.

mobilities, we find $\tau_{\mathrm{mr}} = \frac{m^* \mu_e}{e} \approx 45$ fs, with effective mass $m^* = \frac{\hbar}{u_{\mathrm{F}}} \sqrt{\pi |n_e|} = 0.05 m_e$ for the environmentally-doped monolayer graphene[3] (zero gate voltage). The Fermi velocity is $u_{\mathrm{F}} = 10^6$ m s^{-1} and the measured charge (hole) density is $n_e = -\frac{\epsilon}{ed}\left(V_{\mathrm{g}} - V_{\mathrm{CNP}}\right) = 6 \times 10^{12}$ cm^{-2} at $V_{\mathrm{g}} = 0$ for $V_{\mathrm{CNP}} \approx 9$ V (see main text), where $\epsilon/\epsilon_0 = 3.9$ and $d = 30$ nm are the dielectric constant and thickness, respectively, of the SiO_2 spacer layer in the graphene devices. This corresponds to an environmentally-doped Fermi energy $\varepsilon_{\mathrm{F}} = \pm \hbar u_{\mathrm{F}} (\pi |n_e|)^{\frac{1}{2}} = -300$ meV for these samples.

1.2 Nanoantenna structure and effect of exposed graphene removal

Gold nanostructures are fabricated with 15 nm radius of curvature tips, with good adhesion between the gold and graphene obviating the need for a (highly dissipative) metal adhesion layer. To verify the role of graphene photocurrents in THz generation, we remove the exposed graphene in a metasurface device via 45 s plasma etch (100 W, 10 sccm O_2; Fig. S2a,b). This causes a small

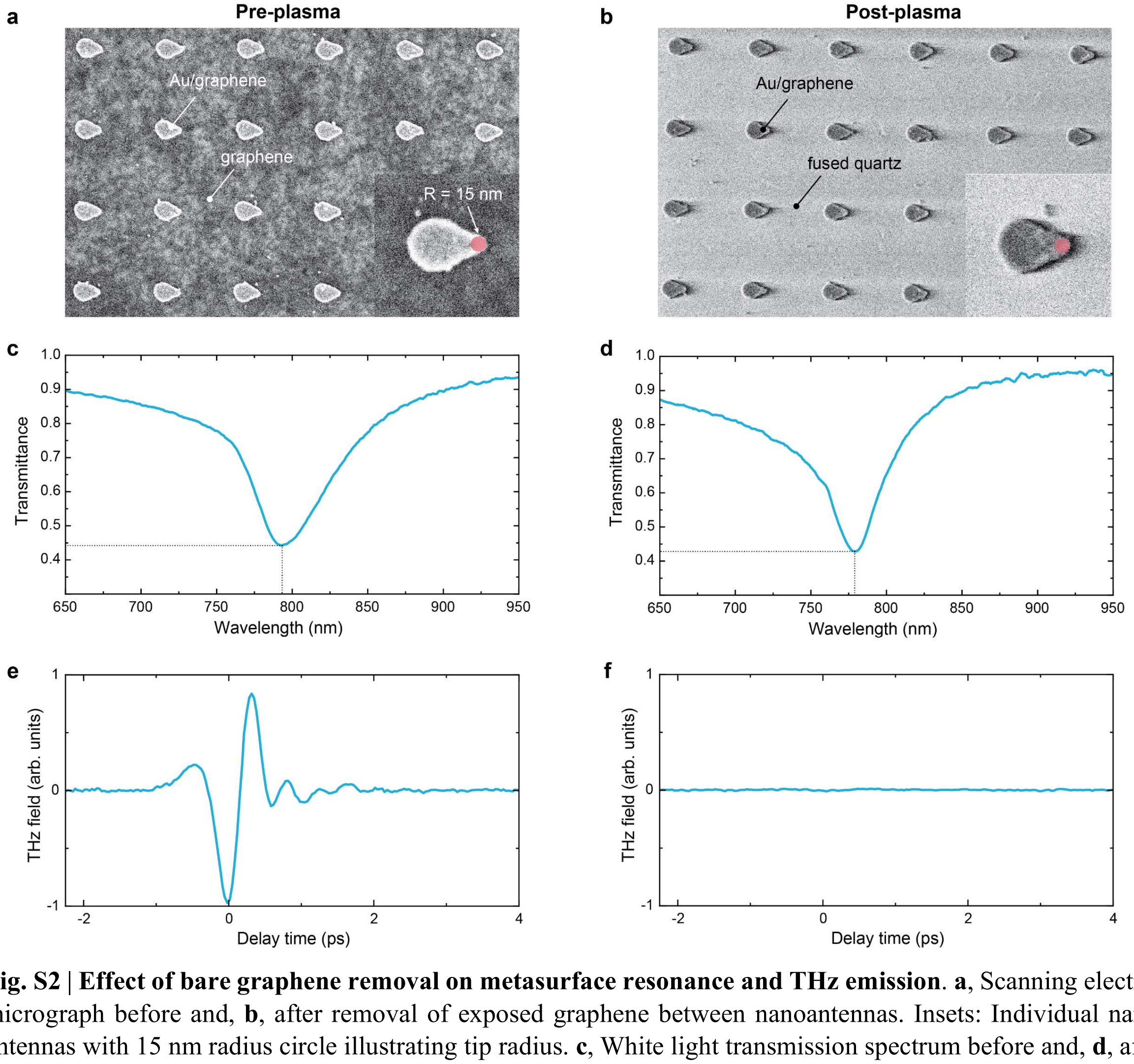

Fig. S2 | Effect of bare graphene removal on metasurface resonance and THz emission. **a**, Scanning electron micrograph before and, **b**, after removal of exposed graphene between nanoantennas. Insets: Individual nano-antennas with 15 nm radius circle illustrating tip radius. **c**, White light transmission spectrum before and, **d**, after graphene removal. **e**, THz time trace before and, **f**, after graphene removal.

blueshift attributed to slight tip deformation during the plasma treatment (Fig. S2c,d) but maintains the nanostructure integrity and strong plasmonic resonance (similar quality factor). Graphene can thus serve as an atomically-thin, minimally-damping adhesion layer between the gold and the fused quartz substrate (or, in general, a variety of materials upon which graphene can be readily transferred). Most importantly for the present studies, the conductivity drops to zero after etching away the bare graphene and no photocurrents are observed. The disappearance of the THz radiation (Fig. S2e,f) under otherwise identical testing conditions illustrates the central role of the graphene photocurrents, as opposed to any local currents or optical rectification within, at the surface of, or beneath the gold nanostructures.

1.3 Spectral characteristics of THz emission

As described in the main text and in further detail below, the photocurrents dynamics are simulated to be very fast, decaying within a couple hundred femtoseconds (Extended Data Fig. 4). This is expected to lead to THz bandwidths exceeding 3 THz, and indeed we find that, in both ZnTe (1 mm thick ⟨110⟩ ZnTe) and photoconductive antenna detections, the THz spectra are limited by the detection response rather than the (metasurface) THz emitters, as summarized in Fig. S3. For the

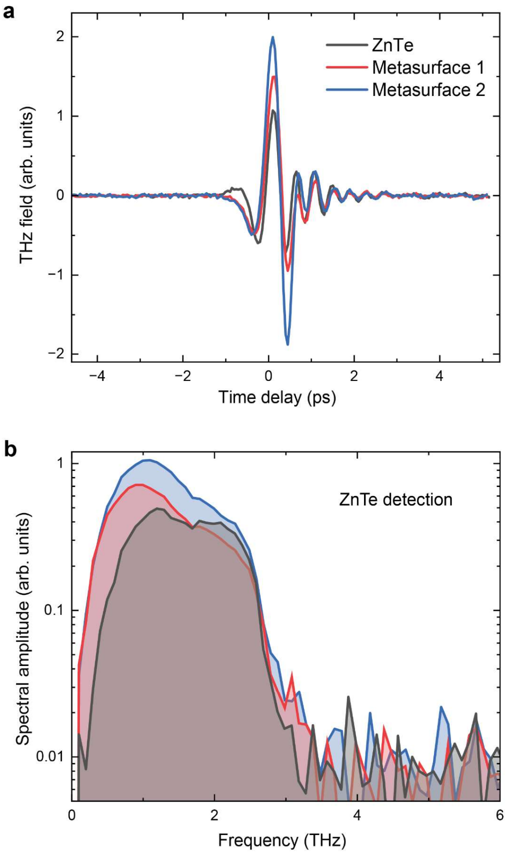

Fig. S3 | Terahertz emission spectra. a, Time traces and **b**, corresponding spectra (via fast Fourier transform) for 1 mm ⟨110⟩ ZnTe source vs. two 800 nm-resonant metasurfaces, measured at 0.3 μJ cm^{-2} incident fluence with 1 mm ⟨110⟩ ZnTe detection. The red dataset is the same as in Fig. 1d of the main text. **c**, Time traces and **d**, corresponding spectra for spintronic emitter vs. 800 nm-resonant metasurface, measured at 1 μJ cm^{-2} incident fluence with photoconductive antenna detection.

ZnTe detection, the bandwidth is limited to ~3 THz due to phase matching[4], with very similar cutoff behavior for both the 800 nm resonant metasurfaces and the 1 mm thick ZnTe source crystal. Both metasurfaces measured here exhibit stronger THz emission than the ZnTe standard. Furthermore, the metasurface emission measured in comparison with a modern ultrabroadband spintronic emitter (TeraSpinTec; nearly flat spectrum out to > 5 THz with 120 fs excitation pulses) is only a factor of ~2 weaker and exhibits a similar spectral width within the detection bandwidth of the photoconductive antenna. We emphasize that this performance comes with very little optimization so far on the metasurface designs (see below). Most important for spectroscopic applications with high-repetition, low-fluence systems is the signal-to-noise ratio, which is comparable for the metasurfaces and standard sources.

Supplementary Note 2: Electromagnetic response

2.1 Preliminary investigation of lattice density and coupling effects

The resonance of plasmonic nanoantennas arranged in a regular array—with spacings comparable to the incident wavelength—is influenced by intermediate-/far-field coupling between resonators. This is known as the surface lattice resonance[5], which can be tuned to influence the resonance wavelength and Q factor compared with isolated nanostructures. Such effects are accounted for here in the electromagnetic simulations via periodic boundary conditions. Rather than iteratively optimize the resonator design, array geometry, and spacing for an 800 nm (or other desired) resonance, we simply optimize the resonator design in a 500 nm square lattice then test

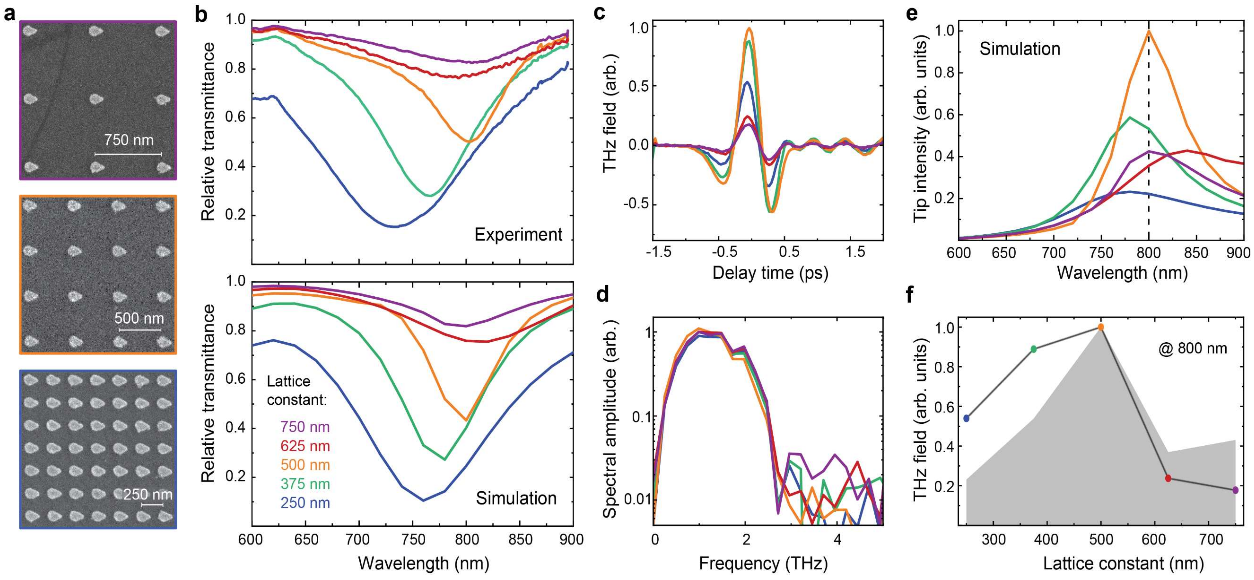

Fig. S4 | Effect of metasurface density. a, Electron micrographs of metasurfaces with 250 nm to 750 nm square lattice constants. **b**, Measured (top) and simulated (bottom) relative transmittance spectra, correcting using the bare graphene/substrate as a reference. **c**, THz time traces and **d**, normalized fast Fourier transformed spectral amplitudes for the different metasurfaces, all measured at 800 nm incident optical wavelength. **e**, Simulated tip hot spot field intensity. **f**, Experimental (solid-dot line) THz field amplitude as a function of lattice constant compared with the simulated value from the density-scaled tip intensity (solid fill).

the effect of varying density (Fig. S4a). A shift in resonance position and quality factor is observed for the varying lattice constants, in good agreement with simulations (Fig. S4b). The 500 nm pitch metasurface exhibits the highest tip intensity enhancement (Fig. S4e) and THz emission (Fig. S4f) and is thus utilized for the present studies of the uniformly-oriented arrays. However, we note that significant further optimization is likely to be possible with different resonator designs, lattices, and nanoantenna spacings.

Because the same 800 nm nanoantenna design is utilized for the Kagome lattice, the surface lattice resonance is not optimized in these systems, leading to the lower field enhancements observed in Fig. 2 of the main text. Nevertheless, the linear response and properties of interest here are not affected. It is important to note that such lattice resonance effects will modify the plasmonic field enhancements but have little effect on the photocurrent distributions beyond the magnitude. In particular, unlike strong near-field coupling effects that can complicate the picture of linear superpositions of individual resonators, this intermediate/far-field coupling between elements of the sub-lattices do not appreciably modify the metasurface linear responses.

In the context of metasurface density effects, we briefly mention the possibility for THz graphene plasmons[6-9]. Such THz plasmons may be launched by the hot-carrier-based currents[10] generated at each nanoantenna. For a specific lattice periodicity, a plasmon mode at a commensurate wavelength can constructively interfere across the metasurface and may enhance the THz radiation via outcoupling through the lattice[11]. However, here we find no spectroscopic evidence of such plasmon modes and, in particular, we observe identical spectra for lattice constants ranging from 250–750 nm (Fig. S4d). The fact that we see no change in the emitted THz radiation with respect to lattice constant provides strong evidence that THz plasmons do not contribute significantly to the response here. Indeed, for these small lattice constants the supported THz plasmon polariton modes are expected to lie beyond 10 THz for graphene on SiO_2 and therefore well outside of our measurement range[6,7]. Nevertheless, the intriguing possibility of generating THz graphene plasmons with incident optical fields (i.e., rectified photocurrents thereof) in similar metasurfaces with appropriate lattice constants, superlattice structure, and/or superposed etched graphene structures warrants further consideration and future investigation.

2.2 Graphene layer dependence

The effect of graphene layer number is tested by comparing the THz emission from metasurfaces fabricated on monolayer, bilayer, 3–5 layer, and 6–8 layer graphene (ACS Material), with SEM micrographs shown in Fig. S5a. An overall decreasing trend of THz emission with layer number is observed (Fig. S5b,c). This can be at least partially explained by increased damping with increase layer number, which leads to less absorbed power per layer (Fig. S5d) and thus less electronic heating, although the total absorbed power across all layers remains similar (Fig. S5e). Furthermore, the effect of metal doping will decrease away from the interfacial layer, leading to less acceleration of the hot carriers excited within deeper layers. Thus, the overall decrease in the current and THz emission signal is expected (simulation result in (Fig. S5c). Nevertheless, further work is needed to understand the various relevant contributions to the layer dependence, as well as ruling out systematics or sample-to-sample variations in the fabrication process for different numbers of layers. The different dispersion around the Dirac point for monolayer versus multilayer graphene are expected to have a small effect here, given the highly environmentally p-doped

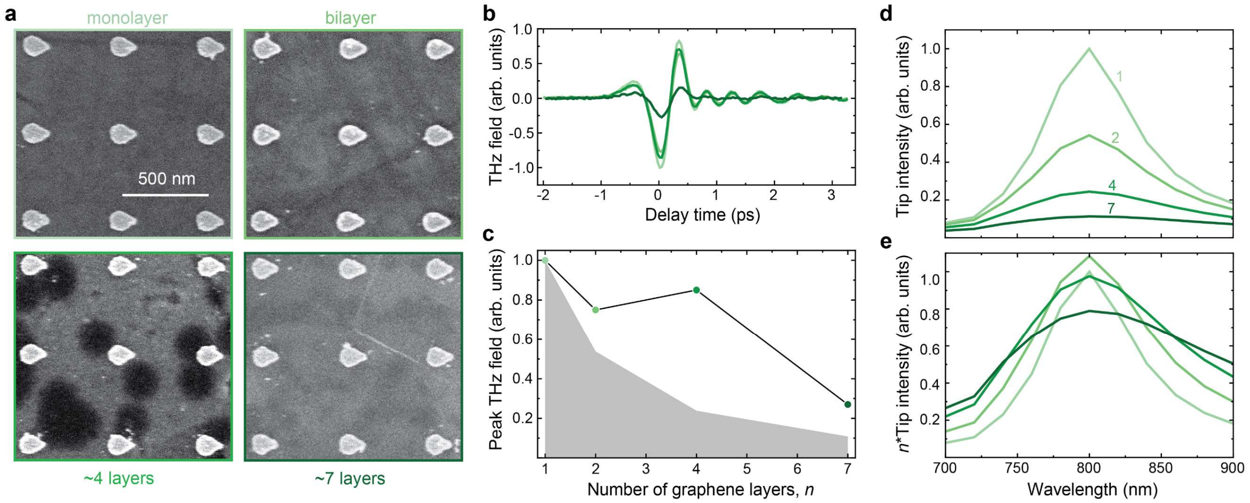

Fig. S5 | Effect of graphene thickness. a, Electron micrographs of metasurfaces fabricated on monolayer, bilayer, 3-5 (~4) layer, and 6-8 (~7) layer graphene. **b**, THz time domain traces for different layer numbers. **c**, Summary of the measured THz field amplitude (dash-dot line) compared with the peak simulated tip field intensity (solid fill) from panel d. **d**, Simulated field intensities at the nanoantenna tip, a few nanometers above the graphene interface. With graphene defined as a boundary layer, the layer number (n) is accounted for via modification of $\tilde{\sigma}_{\mathrm{R}} \rightarrow n\tilde{\sigma}_{\mathrm{R}}$. **e**, Same as panel d, but scaled by n.

graphene (chemical potential far from the Dirac point, well into the Fermi liquid regime) and high photon energies (1.55 eV here). For the present studies, therefore, monolayer graphene appears to be an optimal choice.

Supplementary Note 3: Thermodynamics

3.1 Two-temperature modeling

The full spatiotemporal evolution of electron and lattice temperatures in the metasurface unit cell requires a numerical treatment, as described in the main text (see Fig. 4, Methods, and Extended Data Fig. 5). However, the temperature evolution within bare graphene and individual nanostructures can be treated via simplified two-temperature modeling, neglecting the spatial degrees of freedom, so long as the absorption cross-sections are known. This offers additional insight on the role of the nanolocalized hot spot heating compared with uniform heating due to the 2.3% absorption in bare monolayer graphene. It also offers insight into the internal metal nanostructure heating, and whether these energy sources ought to also be accounted for in the hybrid system. Thus, here we implement the following simplified two-temperature kinetic model for the electronic (T_e) and phonon (T_p) temperatures, evaluated separately for the graphene and gold systems:

$$c_e(T_e)\frac{\partial T_e}{\partial t} = q_{\mathrm{abs}}(t) - g_{\mathrm{er}}\big(T_e - T_p\big), \tag{S1a}$$

$$c_p\frac{\partial T_p}{\partial t} = g_{\mathrm{er}}\big(T_e - T_p\big). \tag{S1b}$$

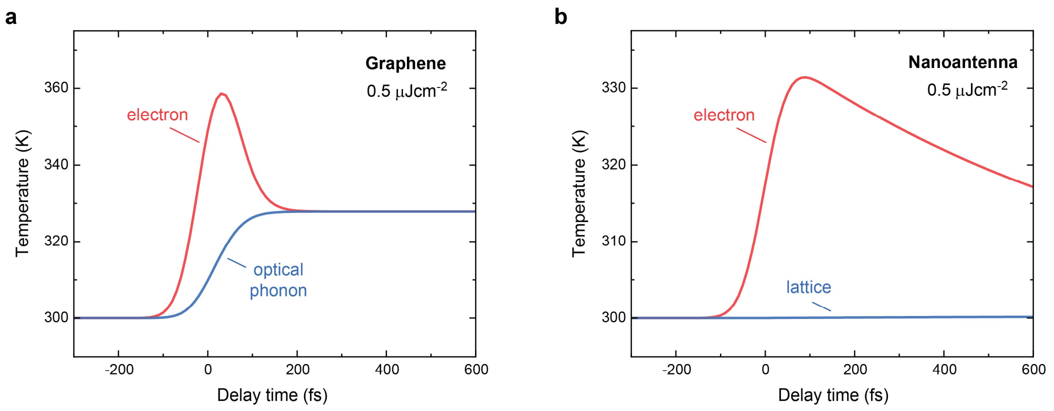

Fig. S6 | Two-temperature modeling for bare graphene and gold nanoantennas. a, Electron and optical phonon temperature evolution for bare graphene (no metasurface) under 100 fs pulse excitation centered around 0 delay time, neglecting acoustic phonon coupling. **b**, Electron and lattice (optical and acoustic phonon) temperature evolution for gold nanoantenna.

Coupling with the substrate is neglected here, and it is approximated that the absorbed power from the optical pulse ($q_{\text{abs}}(t)$) is instantaneously transferred to the thermalized electron gas, bypassing the tens-of-femtosecond electron-electron thermalization times in both systems.

For graphene, we focus only on the energy transfer to the strongly-coupled optical phonons[12], with the electronic specific heat $c_e(T_e)$ described below (note 3.3), absorbed power $q_{\text{abs}}(t) = \frac{1}{2}\sigma_{\text{r}}E(t)^2$ (Methods), energy relaxation coupling constant $g_{\text{er}} = 2 \times 10^7$ WK^{-1}m^{-2} (see note 3.2), and optical phonon specific heat c_p taken from previous work[12]. The results are shown in Fig. S6a for 0.5 μJ cm^{-2} incident fluence and 100 fs pulse duration. Upon comparing the peak $T_e \approx 360$ K with the few-thousand Kelvin peak T_e increase in the hybrid metasurface system (Fig. S9) under the same incident fluence, the role of the concentrated and enhanced power within the plasmonic hot spot in driving the photothermoelectric dynamics is further underscored. Note that at very short times before the excited carrier distribution has thermalized, c_e is not strictly defined, but we maintain the instantaneous carrier–carrier thermalization approximation here. Meanwhile, in both the bare graphene and metasurface systems, the lattice (here meaning strongly-coupled optical phonon) temperature rise is minimal at only a few tens of Kelvin. See note 3.4 for calculations of the time-evolving T_p within the metasurface unit cell.

Now considering only the internal gold nanostructure thermal evolution, the relevant parameters are $c_e = \frac{\pi^2 k_{\text{B}}^2 n_e}{2\varepsilon_{\text{F}}} T_e$ (from Sommerfeld theory; $\varepsilon_{\text{F}} = 5.53$ eV and $n_e = 5.9 \times 10^{22}$ cm^{-3}), $q_{\text{abs}}(t) = \frac{1}{2Z_0 V}\alpha_{\text{abs}}E(t)^2$ (with $Z_0 = 377\ \Omega$ the impedance of free space, $V = 3 \times 10^5$ nm^3 the nanoantenna volume, and $\alpha_{\text{abs}} = 4 \times 10^4$ nm^2 the simulated nanoantenna absorption cross section for resonant 800 nm excitation), $g_{\text{er}} = 2.5 \times 10^{16}$ W K^{-1}m^{-3} is the electron-phonon coupling constant[13], and $c_{\text{l}} = 2.4 \times 10^6$ J K^{-1}m^{-3} for gold. The results are shown in Fig. S6b for 0.5 μJ cm^{-2} incident fluence. For this system, the electronic temperature increase is an even more modest ~30 K and the lattice temperature increase is negligible. Thus, the gold may serve as a thermal sink (unaccounted for in the present work) for the graphene around the tip hot spot, but would not be sufficiently hot to contribute as a source for any of the dynamics.

3.2 Ultrafast dynamics measured via transient reflectivity

We perform transient reflectivity measurements to characterize the coupling constant (g_{er}) between the electronic and lattice systems in our graphene samples. For these measurements we utilize collinear, cross-polarized 800 nm + 800 nm pump-probe microscopy to measure transient (sub-picosecond) changes in the graphene reflectivity. Pump and probe pulses from a Coherent Vitesse oscillator (80 MHz repetition rate) are focused through a Mitutoyo 20× objective onto the sample under high vacuum (~10^{-6} Torr; Janis ST500 optical cryostat). The reflected probe beam is collected on a Si photodiode, with pump filtered out via a linear polarizer. A lock-in amplifier (SR830) is utilized to read out the modulated probe signal induced by the 2.5 kHz-chopped pump beam. The transient reflectivity traces (Fig. S7a) reveal two well-known general behaviors of graphene[14,15]: a fast (~400 fs) decay due to electron–optical phonon scattering and a slower (~1.1 ps) decay due to thermalization with acoustic phonons (acoustic phonon bottleneck). The slower thermalization with the acoustic phonon system can proceed via optical–acoustic phonon–phonon coupling or directly via disorder-assisted electron–acoustic phonon scattering[16].

For suitably high signal-to-noise measurements on the monolayer graphene (with peak $\frac{\Delta R}{R} \sim 0.5 \times 10^{-3}$), we work at high fluences up to 100 μJ cm^{-2}. These values also approach the locally plasmon-enhanced fluences in the metasurface system, but with stronger overall heating without the diffusive cooling pathway. The coupling parameter, g_{er}, is tuned for best agreement between the measured fluence-dependent electron–optical phonon thermalization times and two-temperature models (Fig. S7b). We estimate $g_{\mathrm{er}} \approx 2 \times 10^{7}$ WK^{-1}m^{-2} based on these model assumptions and the high-temperature behavior of graphene discussed in the next section.

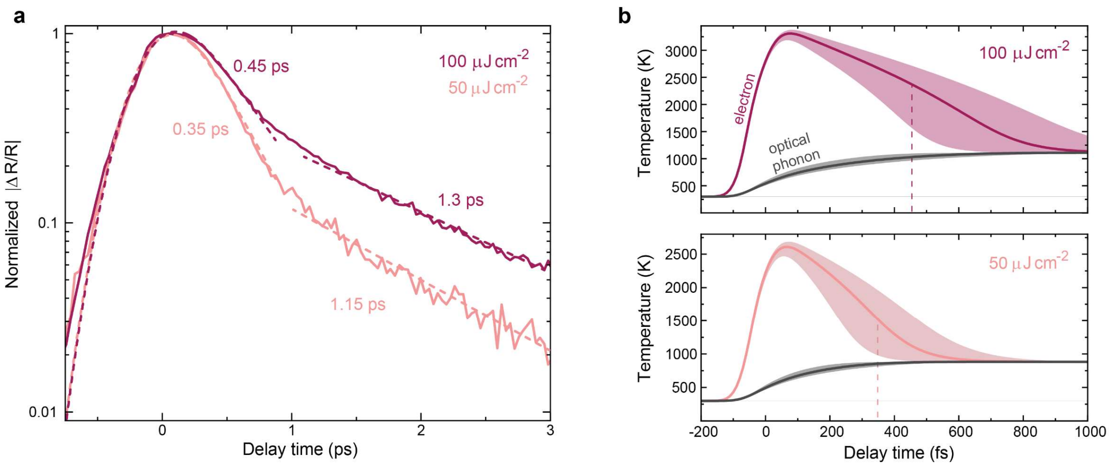

Fig. S7 | Ultrafast transient reflectivity of monolayer graphene and electron-optical phonon coupling constant. a, Transient reflectivity time traces for 50 μJ cm^{-2} and 100 μJ cm^{-2} incident fluence (solid lines). Dashed lines indicate fits to electron-optical phonon thermalization timescales convolved with instrument response function, with separate exponential fits to acoustic phonon thermalization timescales. **b**, Two-temperature modeling for $g_{\mathrm{er}} = 2 \times 10^{7}$ WK^{-1}m^{-2}, with 30% uncertainty bounds from $g_{\mathrm{er}} = 1.3 \times 10^{7}$ to 2.7×10^{7} shown (solid fill). Dashed lines mark the measured decay times.

3.3 High-temperature properties of graphene

Under ultrafast exposure, the electronic system is transiently far from equilibrium with the lattice, reaching temperatures of several thousand Kelvin while the lattice remains near room temperature (thus avoiding issues such as electrode melting that would occur at high temperatures in studies around equilibrium). Expressions commonly utilized at low temperatures or up around room temperature will no longer be valid as $T \rightarrow T_\mathrm{F}$ ($T_\mathrm{F} = 3600$ K for $\varepsilon_\mathrm{F} = -300$ meV). We thus include a discussion on the behavior of several important quantities in the high-temperature regime.

The T_e-dependent electronic specific heat is critical for determining the heating of the electronic system, and can be written generically as

$$c_e(T_e) = \int_{-\infty}^{\infty} d\varepsilon\, \varepsilon\rho(\varepsilon) \frac{\partial f(\varepsilon, T_e)}{\partial T_e}, \tag{S3}$$

where $\rho(\varepsilon) = \frac{2|\varepsilon|}{\pi\hbar^2 u_\mathrm{F}^2}$ is the electronic density of states and $f(\varepsilon, T_e) = \left(e^{(\varepsilon-\mu(T_e))/k_\mathrm{B}T_e} + 1\right)^{-1}$ is the Fermi-Dirac distribution. When the temperature dependence of the chemical potential is accounted for, this c_e interpolates between the Fermi liquid ($k_\mathrm{B}T_e \ll |\mu|$) and Dirac fluid ($k_\mathrm{B}T_e \gg |\mu|$) expressions for the specific heat[17],

$$c_e^\mathrm{FL}(T_e) = \frac{2\pi k_\mathrm{B}^2 \varepsilon_\mathrm{F}}{3\hbar^2 u_\mathrm{F}^2} T_e, \tag{S4a}$$

$$c_e^\mathrm{DF}(T_e) = \frac{18\zeta(3) k_\mathrm{B}^3}{\pi\hbar^2 u_\mathrm{F}^2} T_e^2, \tag{S4b}$$

where $\zeta(3) = 1.202$ is the Reimann-Zeta function. The specific heats in each regime are plotted in Fig. S8a by comparison with the result from Eq. S3. The T_e-dependent chemical potential—which necessarily varies to conserve charge—is given in both regimes by[17,18],

$$\mu^\mathrm{FL}(T_e) = \varepsilon_\mathrm{F}\left(1 - \frac{\pi^2 k_\mathrm{B}^2 T_e^2}{6\varepsilon_\mathrm{F}^2}\right), \tag{S5a}$$

$$\mu^\mathrm{DF}(T_e) = \frac{\varepsilon_\mathrm{F}^2}{4\ln(2) k_\mathrm{B} T_e}, \tag{S5b}$$

and here we simply interpolate between these two limits (Fig. S8a) as

$$\mu(T_e) \approx \left(1 + \frac{T_e}{T_\mathrm{F}/2}\right)^{-6} \mu^\mathrm{FL} + \left(1 - \left(1 + \frac{T_e}{T_\mathrm{F}/2}\right)^{-6}\right) \mu^\mathrm{DF}, \tag{S6}$$

positioned around $T_\mathrm{F}/2$ (half the Fermi temperature, $T_\mathrm{F} = \varepsilon_\mathrm{F}/k_\mathrm{B}$) to preclude the sign change that occurs in Eq. S5a as $T_e \rightarrow T_\mathrm{F}$.

The electron-electron scattering rate (τ_{ee}^{-1}) in graphene in the Fermi liquid regime scales as $-T_e^2 \ln(\frac{k_\mathrm{B}T_e}{|\varepsilon_\mathrm{F}|})$ at low temperatures[19] ($T_e \ll T_\mathrm{F}$), but this expression becomes problematic as $T_e \rightarrow T_\mathrm{F}$ ($\tau_{ee}^{-1} \rightarrow 0$ and then becomes negative). The high-temperature behavior can be evaluated using Fermi's golden rule[20],

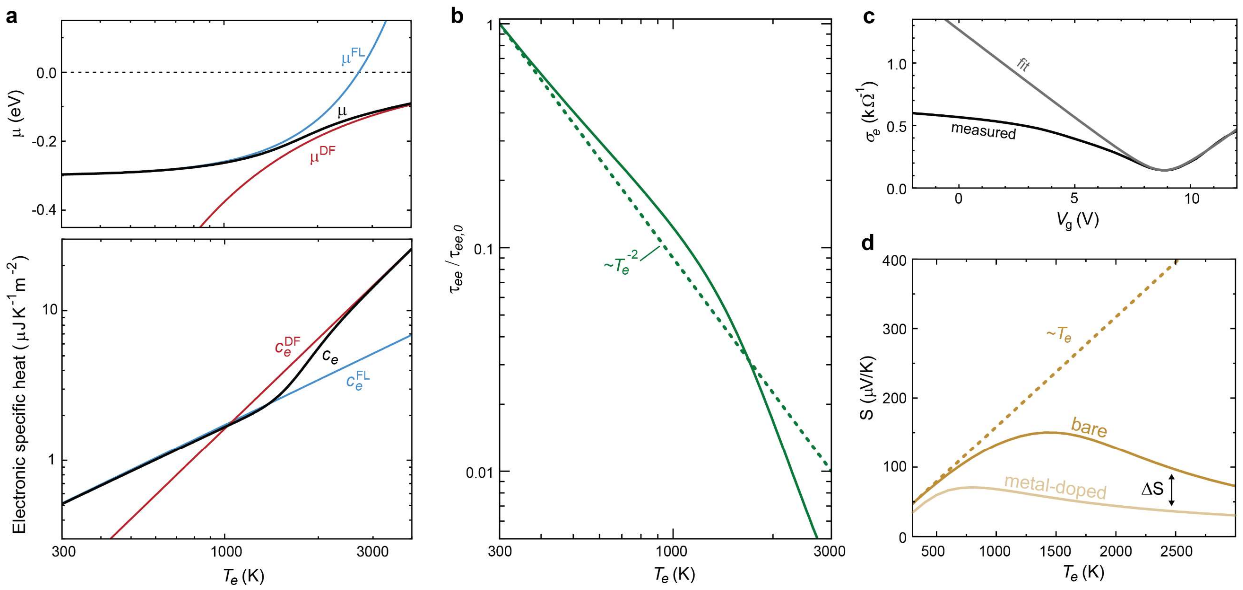

Fig. S8 | High-T_e calculations for graphene. **a**, Chemical potential (top) and specific heat (bottom). **b**, Scattering time relative to T_0 value compared with standard T_e^{-2} Fermi liquid theory behavior. **c**, Measured conductivity for graphene device and idealized fit excluding extrinsic effects, utilized for calculations of the Seebeck coefficient. **d**, Seebeck coefficient calculated for the bare and metal-doped graphene regions based on Eq. S9, compared with the T_e-linear behavior that prevails at low temperatures.

$$\tau_{ee}^{-1}(\varepsilon_i, T_e) = \frac{2\pi}{\hbar} M^2 \int_{-\infty}^{\varepsilon_i} d\varepsilon_f \left(1 - f(\varepsilon_f)\right) \rho(\varepsilon_f)$$
$$\times \int_{-\infty}^{\infty} d\varepsilon_i' \, f(\varepsilon_i') \rho(\varepsilon_i') \big(1 - f(\varepsilon_i' + \Delta)\big) \rho(\varepsilon_i' + \Delta) \,, \qquad \text{(S7)}$$

where $\Delta = \varepsilon_i - \varepsilon_f$, M is the transition matrix element (approximated as constant), subscripts indicate initial (i) and final (f) states, and the primed (unprimed) energies refer to the secondary (primary) electrons. The approximation of constant M neglects the onset of interband transitions at high temperatures, but this simplifying assumption is suitable for the purposes of the present discussion. Integration of Eq. S7 yields a nearly quadratic energy dependence for the Fermi liquid, $\tau_{ee}^{-1} \sim (\varepsilon - \varepsilon_{\mathrm{F}})^2$, modified at low energies ($|\varepsilon_i - \varepsilon_{\mathrm{F}}| < k_{\mathrm{B}} T_e$) due to the finite temperature. The overall T_e dependence is then determined by averaging over energy,

$$\tau_{ee}^{-1}(T_e) = \int_{-\infty}^{\infty} d\varepsilon_i \, \tau_{ee}^{-1}(\varepsilon_i, T_e) \frac{\partial f}{\partial \varepsilon_i} \,. \qquad \text{(S8)}$$

The result is plotted in Fig. S8b, revealing $\tau_{ee}^{-1}(T_e) \sim T_e^2$ dependence. This behavior is responsible for locally driving the electronic system around the nanoantennas into an apparently hydrodynamic regime, where $\tau_{ee} \ll \tau_{\mathrm{mr}}$, influencing the spatially-varying viscosity, $\nu \sim \tau_{ee}$. The room temperature value of τ_{ee} was determined to be ~200 fs in previous work[21].

The Seebeck coefficient at high temperatures can be determined from the generalized form of the Mott relation[22,23],

$$S(\mu) = -\frac{1}{eT_e}\frac{\int_{-\infty}^{\infty} d\varepsilon\, (\varepsilon-\mu)\sigma_e(\varepsilon)\frac{\partial f}{\partial \varepsilon}}{\int_{-\infty}^{\infty} d\varepsilon\, \sigma(\varepsilon)\frac{\partial f}{\partial \varepsilon}}, \tag{S9}$$

The measured electrical conductivity is fit to an idealized functional form[24,25], $\sigma_e(\mu) = \sigma_{\min}\left(1+\frac{\mu^4}{w^4}\right)^{-2}$ where $\sigma_{\min}$ is the minimum conductivity and $w = 100$ meV is the width of the charge neutrality region (Fig. S8c). This removes extrinsic contributions that do not influence the local conductivity at the nanoantenna tips. We approximate σ_e (which also appears in the Wiedemann-Franz expression for κ_e) to be independent of T_e, as Umklapp scattering is suppressed in graphene. At the high temperatures relevant to the present studies, Eq. S9 yields a sublinear and even nonmonotonic dependence on T_e, plotted in Fig. S8d for both the bare and gold-doped graphene regions. For the bare graphene, Eq. 6 (Fig. S8a) is utilized for $\mu_{\text{bare}}(T_e)$, while we approximate $\mu_{\text{pinned}} \approx -50$ meV as a constant pinned by the gold chemical potential. The resulting $\Delta S(T_e)$ is then responsible for the photothermoelectric acceleration of charge.

3.4 Thermodynamic energy flow

The energy cascade in graphene following ultrafast optical excitation generally involves as (i) rapid carrier-carrier thermalization within $\sim$100 fs, (ii) thermalization with optical phonons within a few hundred femtoseconds, and (iii) full thermalization with acoustic phonons on $>$ 1 ps timescales[14,15,17,18,26,27]. As mentioned above, thermalization with the acoustic phonon system can be accelerated via a disorder-assisted (so-called supercollision) cooling channel, directly between the electrons and acoustic phonons[16,17]. If photon energy $> 2\varepsilon_{\text{F}}$ such that interband transitions are allowed (as is the case here), separated Fermi-Dirac distributions for the electron and hole gases can be established on tens-of-femtosecond timescales before a single electronic Fermi-Dirac distribution is established on $<$ 150 fs timescales[18,26]. It should be noted that the tens-of-femtosecond timescale for internal thermalization of the athermal excited carriers is different from τ_{ee} of the thermalized distribution ($\sim$200 fs at room temperature, down to a few femtoseconds at 3000 K). Here we approximate an instantaneous transfer of energy between the optical pulse and the fully thermalized electronic distribution. While the convolution of these $\sim$100 fs dynamics with the $\sim$100 fs optical pulse duration can be expected to skew the short-timescale behaviors, such effects will have little bearing on the overall thermodynamic energy and hydrodynamic momentum flows examined here.

The cooling length of the superheated electrons is estimated to be $\sim$150 nm here (Methods), which is much shorter than the gap between structures ($>$ 300 nm for 500 nm lattice constant). While we see that surface lattice resonance effects appear to be dominant in density-dependence studies (Supplementary Note 2), the cooling length may also play an important role when the inter-nanoantenna gap size becomes short enough. In particular, the electronic thermal field from one tip can interact with the back side of the adjacent nanoantenna, leading to the opposite junction photothermoelectric effect and partial cancellation of the currents. With more complex spatial arrangements of the structures, such effects can indeed serve to enrich the spatiotemporal energy

and momentum flows in these systems (introducing, for instance, a phase delay between the forward and backward contributions due to finite time spreading of the thermal field).

In some previous works[12,26], the strong coupling between the electronic and optical phonon systems has led to rapid optical phonon heating up to > 1000 K. These works, however, employ 30–300-fold higher fluences than typically utilized in the present studies (< 1 μJ cm^{-2}) on bare graphene, consistent with the results shown in Fig. S7. The total absorbed power in the metasurfaces is relatively modest, with electronic superheating only occurring due to the highly concentrated plasmonic field distribution. As a result of this relatively low overall heat load, fast electronic thermal diffusion, and the delayed energy transfer to the optical phonon system, the peak optical phonon temperature within the unit cell is calculated to be merely 390 K (Fig. S9). Coupling to the acoustic phonon system on longer timescales is neglected here. The strongly heated electronic system (with dramatically faster τ_{ee}) and near-room-temperature phonon system (with little change to τ_{mr}) thus supports the view of a transient light-induced hydrodynamic phase. The resulting charge flows are discussed in the main text and examined further in the next section.

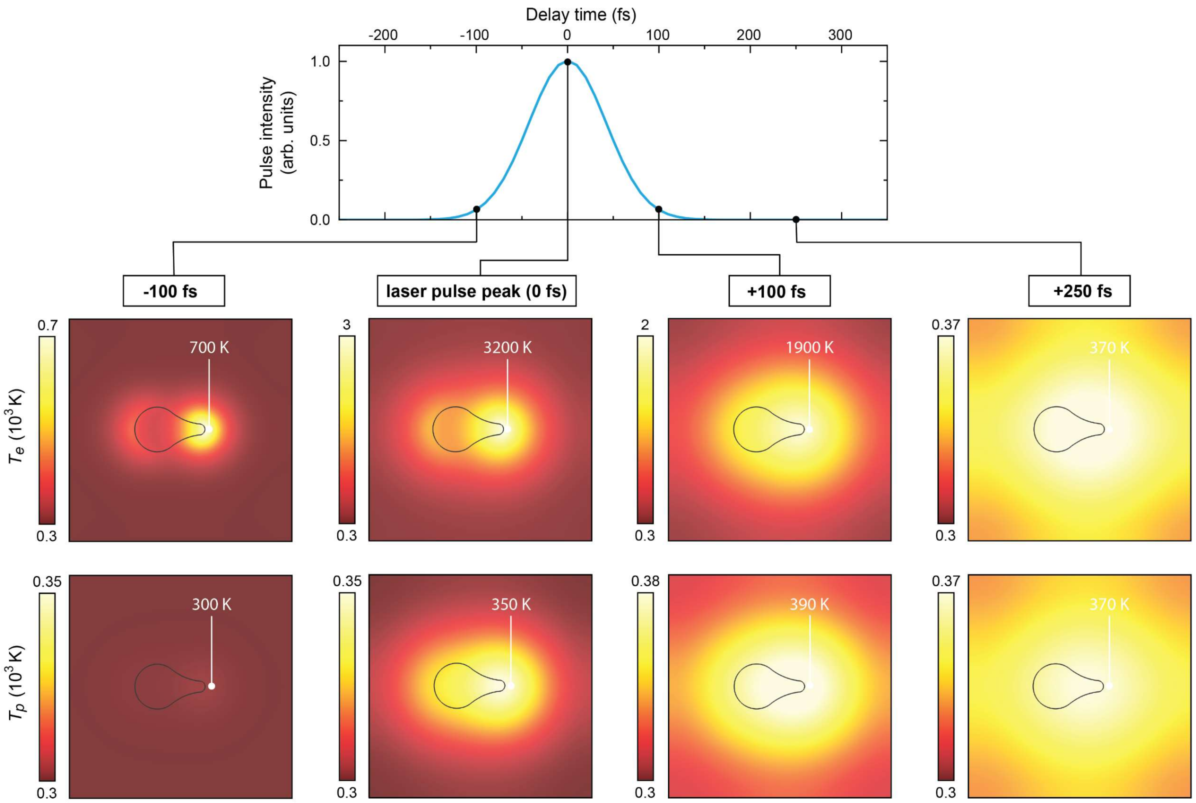

Fig. S9 | Electronic and optical phonon temperature evolution in graphene. Spatial temperature distributions at various times relative to the incident laser pulse, assuming instantaneous transfer of energy into a locally thermalized hot carrier distribution.

Supplementary Note 4: Hydrodynamics

4.1 Quantitative and qualitative comparison with experiments

Quantitative results for simulated DC photocurrents (averaging the ultrafast time-evolving current flow) are shown as a function of incident laser fluence in Fig. S10. Comparison with experimental values shows good agreement, to within a factor of 2. Note that the applicability of the hydrodynamic description breaks down at low incident fluences due to insufficient electronic heating. Sub-linear behavior observed at fluences approaching 1 μJ cm^{-2} in both the simulations and experiments is due to the photothermal process, involving different nonlinear contributions of the electron temperature (e.g., Fig. S8). To study the damage threshold of these metasurfaces where transient heating during a single pulse is sufficient to melt and/or delaminate the structures, we employ a 1 kHz amplified laser operating at 800 nm. While THz emission is observed in this system using ZnTe electro-optic sampling, we do not report the fluence dependence in this high-fluence range as more careful studies are required to preclude competing degradative effects, such as graphene oxidation. Nevertheless, we see a clear transition from regular THz time traces to pathological behavior around 100 μJ cm^{-2}, with SEM imaging confirming metasurface damage around these fluences (Fig. S10b, inset). We estimate gold lattice heating around ~50 K at this fluence, which may be locally hotter within the tip region and graphene interface. Extending the hydrodynamic modeling out to this range using $c_e(T_e)$ shown in Fig. S8, we approximate the high-fluence dependence of the system accounting for local (plasmonic-field-enhanced) saturable absorption of the graphene[28] with a saturation fluence around 650 μJ cm^{-2}. Strongly sub-linear behavior is observed beyond ~1 μJ cm^{-2} due both to the saturable absorption as well as nonlinear thermal effects. These high-fluence calculations should, however, be viewed only qualitatively due to unphysically high electron temperatures (approaching 10^4 K) modeled within this regime, at which point effects such as thermionic emission from the graphene are expected to significantly alter the dynamics. A higher degree of linearity may be expected up to higher fluences in optoelectronic metasurfaces composed of other materials where non-photothermoelectric mechanisms prevail. Furthermore, the damage threshold may be extended even in the present

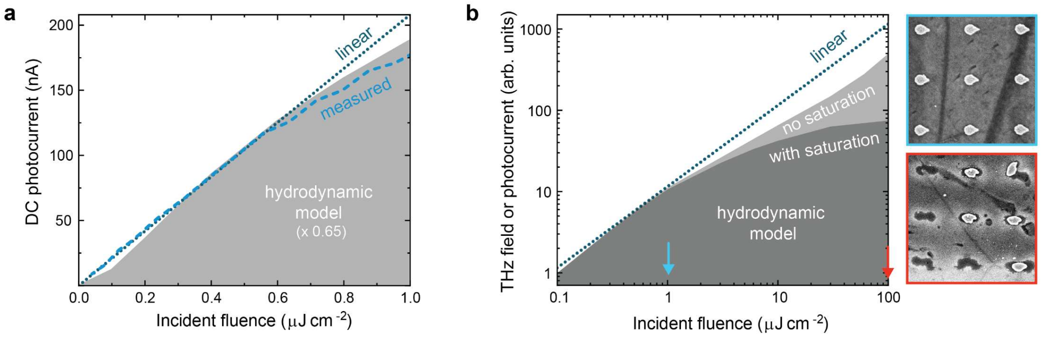

Fig. S10 | Incident fluence dependence. a, Measured fluence-dependent DC photocurrent (dashed blue line) versus values calculated via hydrodynamic model (solid gray fill). The dotted line shows a best-fit linear dependence to the experimental data at low fluence. **b**, Simulated extended fluence dependence via hydrodynamic model, with and without graphene saturable absorption taken into account. Insets: SEM micrographs collected on representative metasurface regions after 1 μJ cm^{-2} laser exposure (top) and after 100 μJ cm^{-2} laser exposure (bottom) using a 1 kHz amplified laser.

systems with future investigations of protective dielectric coatings[29], graphene encapsulation, and/or a different choice of substrate. Other refractory plasmonic materials, such as TiN and conducting oxides, could also be used in place of noble metals to operate at higher excitation fluences at infrared wavelengths.

The major sources of uncertainty in the model include the following: (i) The precise plasmonic field enhancement within the graphene layer, due to challenges in classical (and idealized) simulations of interfaces. (ii) The spatial extent of the transition region between the metal-doped and bare graphene regions, which is taken here to be ~10 nm via Gaussian convolution over the two domains. (iii) The effects of nonuniform charge density. (iv) High-temperature behaviors in the thermodynamic and hydrodynamic parameters. Despite these sources of uncertainty, the simulated flow behaviors remain robust to changes in the exact functional forms

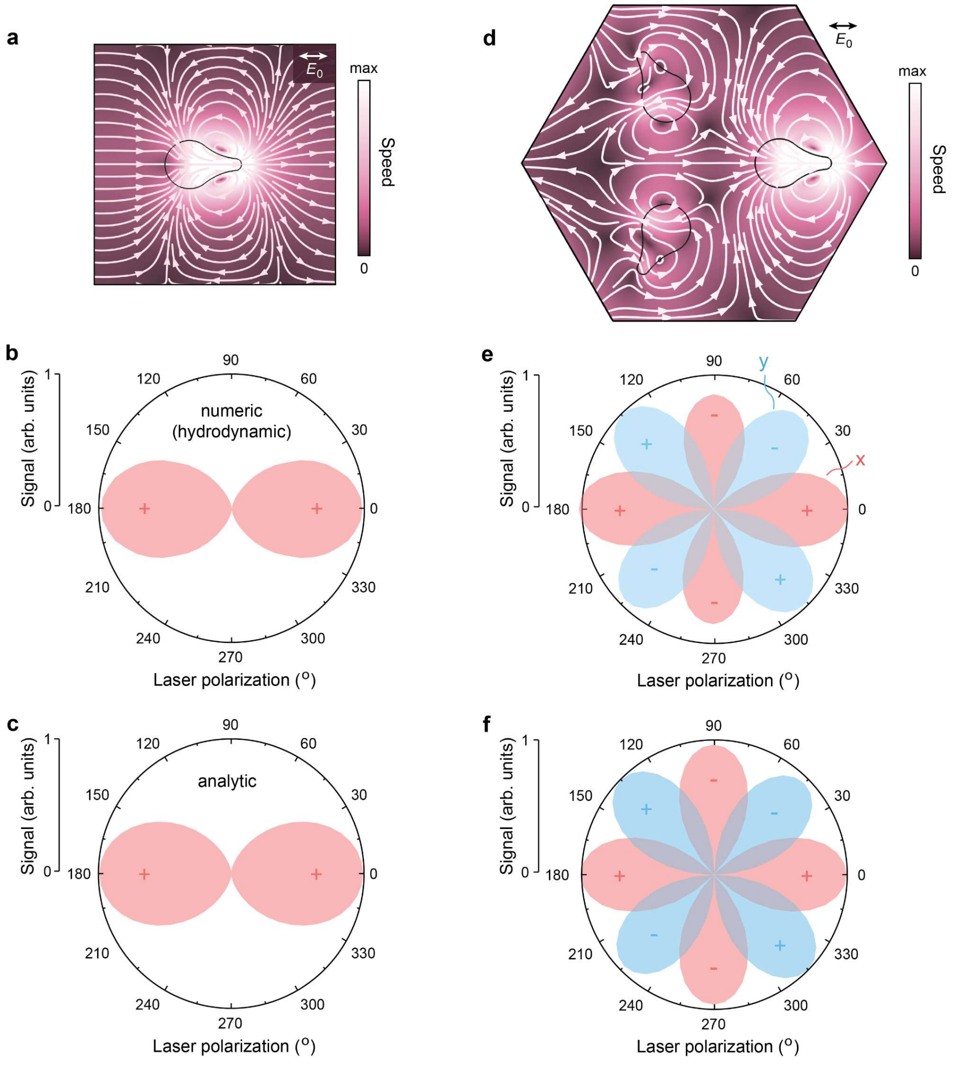

Fig. S11 | Comparison between analytic and hydrodynamic polarization dependence. a, Flow profile for the unit cell of the square lattice. **b**, Polarization dependence of the photocurrent (x component; y component is negligibly small) calculated via hydrodynamic model. **c**, Polarization dependence of the photocurrent calculated analytically for a linear response (x component; y component is identically zero by symmetry). **d–f**, Same as panels a–c but for the Kagome lattice unit cell (now including y photocurrent component).

and values of the heat capacities, thermal conductivities, thermopower, etc. Therefore, the good agreement between experiment and modeling—based on the best approximations described above and using no ad hoc parameter variation/optimization—suggests that such simulations are a reasonable starting point for understanding and predicting the nanoscale flow behaviors occurring in our system.

Calculated polarization dependence is also in good agreement with experiments (and the analysis based on a simple linear response), as shown in Fig. S11. Compared with the simple linear responses and superposition of currents from the different nanoantennas described in the main text, the hydrodynamic simulations also account for coupling that occurs due to the incompressibility constraint. Even so, the symmetry of the light-matter interaction evidently prevails.

4.2 Chirality and magnetization

Most of the metasurfaces studied here have been achiral, with the exception of the perturbed Kagome lattice (Extended Data Fig. 2) with local planar chirality, and the azimuthal metasurface (Fig. 3) with global planar chirality. Although transient spatially-varying magnetic fields will accompany all of the current flow profiles, the introduction of structural chirality (i.e., with no mirror symmetry planes perpendicular to the metasurface) introduces chiral orbital nanocurrents and net orbital magnetization. A simulated example of this is shown in Fig. S12. Such systems may prove useful for controlling magnetic interactions within various symmetry-broken metasurfaces or in nearby materials, although a simple estimate limits the transient magnetic fields to the μT or low-mT ranges in graphene. This limit is imposed by the current density, $\mathbf{j} = en_e\mathbf{u}$, where $u < u_\mathrm{F}$ (and $\ll u_\mathrm{F}$ under most experimentally accessible conditions) and $n_e \lesssim 10^{13}$ cm^{-2} by electrostatic doping.

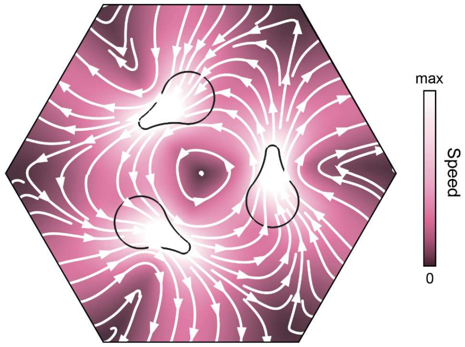

Fig. S12 | Local vortical flow and net magnetization in a chiral unit cell. An example of a chiral nanostructure arrangement with corresponding counter-clockwise hydrodynamic flow, serving as a local magnetic dipole.

Supplementary References

1 Li, X. S. *et al.* Large-area synthesis of high-quality and uniform graphene films on copper foils. *Science* **324**, 1312-1314 (2009).

2 Wang, H. M., Wu, Y. H., Cong, C. X., Shang, J. Z. & Yu, T. Hysteresis of electronic transport in graphene transistors. *ACS Nano* **4**, 7221-7228 (2010).

3 Novoselov, K. S. *et al.* Two-dimensional gas of massless Dirac fermions in graphene. *Nature* **438**, 197-200 (2005).

4 Nahata, A., Weling, A. S. & Heinz, T. F. A wideband coherent terahertz spectroscopy system using optical rectification and electro-optic sampling. *Appl. Phys. Lett.* **69**, 2321-2323 (1996).

5 Kravets, V. G., Kabashin, A. V., Barnes, W. L. & Grigorenko, A. N. Plasmonic surface lattice resonances: A review of properties and applications. *Chem. Rev.* **118**, 5912-5951 (2018).

6 Fei, Z. *et al.* Infrared nanoscopy of Dirac plasmons at the graphene-SiO_2 interface. *Nano Lett.* **11**, 4701-4705 (2011).

7 Ju, L. *et al.* Graphene plasmonics for tunable terahertz metamaterials. *Nat. Nanotechnol.* **6**, 630-634 (2011).

8 Alonso-González, P. *et al.* Acoustic terahertz graphene plasmons revealed by photocurrent nanoscopy. *Nat. Nanotechnol.* **12**, 31-35 (2017).

9 Koppens, F. H. L., Chang, D. E. & de Abajo, F. J. G. Graphene plasmonics: A platform for strong light-matter interactions. *Nano Lett.* **11**, 3370-3377 (2011).

10 Li, Y. Y., Ferreyra, P., Swan, A. K. & Paiella, R. Current-driven terahertz light emission from graphene plasmonic oscillations. *ACS Photonics* **6**, 2562-2569 (2019).

11 Okisu, N., Sambe, Y. & Kobayashi, T. Far-infrared emission from two-dimensional plasmons in AlGaAs/GaAs heterointerfaces. *Appl. Phys. Lett.* **48**, 776-778 (1986).

12 Lui, C. H., Mak, K. F., Shan, J. & Heinz, T. F. Ultrafast photoluminescence from graphene. *Phys. Rev. Lett.* **105**, 127404 (2010).

13 Groeneveld, R. H. M., Sprik, R. & Lagendijk, A. Femtosecond spectroscopy of electron-electron and electron-phonon energy relaxation in Ag and Au. *Phys. Rev. B* **51**, 11433-11445 (1995).

14 Breusing, M. *et al.* Ultrafast nonequilibrium carrier dynamics in a single graphene layer. *Phys. Rev. B* **83**, 153410 (2011).

15 Wang, H. N. *et al.* Ultrafast relaxation dynamics of hot optical phonons in graphene. *Appl. Phys. Lett.* **96**, 081917 (2010).

16 Song, J. C. W., Reizer, M. Y. & Levitov, L. S. Disorder-assisted electron-phonon scattering and cooling pathways in graphene. *Phys. Rev. Lett.* **109**, 106602 (2012).

17 Massicotte, M., Soavi, G., Principi, A. & Tielrooij, K. J. Hot carriers in graphene - fundamentals and applications. *Nanoscale* **13**, 8376-8411 (2021).

18 Gierz, I. *et al.* Snapshots of non-equilibrium Dirac carrier distributions in graphene. *Nat. Mater.* **12**, 1119-1124 (2013).

19 Giuliani, G. F. & Quinn, J. J. Lifetime of a quasiparticle in a two-dimensional electron-gas. *Phys. Rev. B* **26**, 4421-4429 (1982).

20 Zarate, E., Apell, P. & Echenique, P. M. Calculation of low-energy-electron lifetimes. *Phys. Rev. B* **60**, 2326-2332 (1999).

21 Bandurin, D. A. *et al.* Negative local resistance caused by viscous electron backflow in graphene. *Science* **351**, 1055-1058 (2016).

22 Ashcroft, N. W. & Mermin, N. D. *Solid state physics*. (Saunders College, 1976).

23 Ghahari, F. *et al.* Enhanced thermoelectric power in graphene: Violation of the Mott relation by inelastic scattering. *Phys. Rev. Lett.* **116**, 136802 (2016).

24 Shiue, R. J. *et al.* High-responsivity graphene-boron nitride photodetector and autocorrelator in a silicon photonic integrated circuit. *Nano Lett.* **15**, 7288-7293 (2015).

25 Shautsova, V. *et al.* Plasmon induced thermoelectric effect in graphene. *Nat. Commun.* **9**, 5190 (2018).

26 Johannsen, J. C. *et al.* Direct view of hot carrier dynamics in graphene. *Phys. Rev. Lett.* **111**, 027403 (2013).

27 Brida, D. *et al.* Ultrafast collinear scattering and carrier multiplication in graphene. *Nat. Commun.* **4**, 1987 (2013).

28 Winzer, T. *et al.* Absorption saturation in optically excited graphene. *Appl. Phys. Lett.* **101**, 221115 (2012).

29 Albrecht, W. *et al.* Single particle deformation and analysis of silica-coated gold nanorods before and after femtosecond laser pulse excitation. *Nano Lett.* **16**, 1818-1825 (2016).
